# An enzyme-activatable and cell-permeable Mn^III^-porphyrin as a highly efficient *T*_1_ MRI contrast agent for cell labeling†

**DOI:** 10.1039/c5sc04252f

**Published:** 2016-03-16

**Authors:** Inga E. Haedicke, Tan Li, Yong Le K. Zhu, Francisco Martinez, Amanda M. Hamilton, Donna H. Murrell, Joris T. Nofiele, Hai-Ling M. Cheng, Timothy J. Scholl, Paula J. Foster, Xiao-an Zhang

**Affiliations:** a Department of Chemistry, University of Toronto Toronto ON M5S 3H6 Canada xiaoan.zhang@utoronto.ca; b Department of Physical and Environmental Sciences, University of Toronto Scarborough 1265 Military Trail Toronto ON M1C 1A4 Canada; c Department of Biological Sciences, University of Toronto Scarborough 1265 Military Trail Toronto ON M1C 1A4 Canada; d Imaging Research Laboratories, Robarts Research Institute 1151 Richmond St. N London ON N6A 5B7 Canada tscholl@robarts.ca pfoster@robarts.ca; e Department of Medical Biophysics, Western University 1151 Richmond St. N N6A 5C1 London Ontario Canada; f Physiology & Experimental Medicine, The Research Institute, Hospital for Sick Children Toronto Ontario Canada M5G 1X8; g Translational Biology & Engineering Program, Ted Rogers Centre for Heart Research, University of Toronto Toronto Ontario Canada M5S 3G9; h The Edward S. Rogers Sr. Department of Electrical & Computer Engineering, University of Toronto Toronto Ontario Canada M5S 3G9; i Institute of Biomaterials and Biomedical Engineering, University of Toronto Toronto Ontario Canada M5S 3G9 hailing.cheng@utoronto.ca

## Abstract

Magnetic resonance imaging (MRI) is a preferred technique for noninvasively monitoring the fate of implanted cells, such as stem cells and immune cells *in vivo*. Cellular MRI requires contrast agents (CAs) to label the cells of interest. Despite promising progress made in this emerging field, highly sensitive, stable and biocompatible *T*_1_ CAs with high cell permeability and specificity remains an unmet challenge. To address this need, a novel Mn^III^-porphyrin, MnAMP was designed and synthesized based on the modification of Mn^III^tetra(carboxy-porphyrin) (MnTCP), a small and highly stable non-Gd extracellular CA with good biocompatibility and high *T*_1_ relaxivity (*r*_1_ = 7.9 mM^−1^ s^−1^) at clinical field of 3 Tesla (T). Cell permeability was achieved by masking the polar carboxylates of MnTCP with acetoxymethyl-ester (AM) groups, which are susceptible to hydrolysis by intracellular esterases. The enzymatic cleavage of AM groups led to disaggregation of the hydrophobic MnAMP, releasing activated MnTCP with significant increase in *T*_1_ relaxivity. Cell uptake of MnAMP is highly efficient as tested on two non-phagocytic human cell lines with no side effects observed on cell viability. MRI of labeled cells exhibited significant contrast enhancement with a short *T*_1_ of 161 ms at 3 T, even though a relatively low concentration of MnAMP and short incubation time was applied for cell labeling. Overall, MnAMP is among the most efficient *T*_1_ cell labeling agents developed for cellular MRI.

## Abstract 2

MnAMP, a cell-trappable pro-contrast agent gets enzymatically activated and accumulated intracellularly to provide a strong MRI signal for cell labeling.

## Graphical abstract

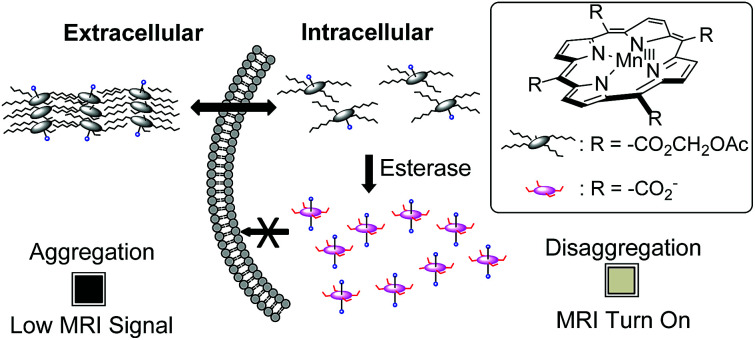

## Introduction

Our capability to study and utilize cell functions has greatly increased owing to advances in cellular imaging techniques. In recent years, as the research field involving cell transplantation, such as adoptive immunotherapy^1^ and stem-cell therapy,^2,3^ is rapidly growing, there are increasing demands for new translational imaging methods to noninvasively label and track implanted cells *in vivo*.^4,5^ Among conventional clinical imaging modalities, MRI has a unique combination of advantages, including true non-invasiveness, deep tissue penetration, intrinsic anatomic information with delicate tissue contrast, a large and adjustable field of view (FOV) with sub-millimeter resolution, and an unlimited time window for repeated imaging. Therefore, MRI has become a preferred choice for monitoring spatial and temporal changes in cell localization and distribution *in vivo*. Since the native relaxation times (*T*_1_ and *T*_2_) of different cell types are too similar to detect by MRI, a group of implanted cells can only be visualized after labeling *in vitro* prior to transplantation *in vivo*.^6^ Cells have been successfully labeled with traditional CAs acting on ^1^H-NMR relaxation enhancement or using non-proton ^19^F agents^7–9^ without background signal. Currently the most widely used CAs for MR cell imaging are *T*_2_ agents based on superparamagnetic iron oxide nanoparticles (SPIOs), owing to high sensitivity down to single cell detection^10^ and adjustable cell-permeability, which has been demonstrated in a variety of both animal studies and clinical trials.^11,12^ There are however limitations inherent in the contrast mechanism utilized by these agents. The negative contrast enhancement (image darkening) generated by *T*_2_ agents can also result from different sources, including tissues with high iron content, hemorrhages, hemochromatosis or other artefacts associated with magnetic susceptibility such as air spaces and tissue interfaces.^13,14^*T*_1_ CAs can overcome these limitations by providing conspicuous positive contrast enhancement (brightening) on MR images, more specific to distinctly labeled cells. In fact, Gd-based *T*_1_ CAs (GBCAs) dominate the regular clinical applications of contrast enhanced MRI.^15^ While most clinical GBCAs belong to extracellular fluid (EFC) agents considered to be cell-impermeable, a study led by Aime *et al.* demonstrated MRI-observable cellular uptake of Gd-HPDO3A, presumably *via* pinocytosis. High concentrations (5–100 mM) and long labeling times (12–24 h), however, were required for sufficient cell labeling, due to low permeability.^16^ A more sophisticated approach is to incorporate multiple Gd-complexes into nanocarriers,^17^ such as liposomes,^18^ virus capsids,^19,20^ protein cargos^21^ or glucan particles^22^ to improve relaxivity and cell loading capacity. In addition, attachment of small molecular Gd-chelates or Gd-nanocarriers with cell penetrating peptides^23–26^ or lipophilic moieties^27,28^ can enhance the cell uptake. While more stable cyclic Gd-chelates are the preferred choice for cell labeling, long-term intracellular stability of GBCAs is still a concern due to toxicity of free Gd ions.^29^ Another limitation of typical GBCAs is that *T*_1_ relaxivity (*r*_1_) is not optimal, especially at high field, a favorable condition for cellular MRI. Quenching of *r*_1_ down to below 1 mmol^−1^ s^−1^ was observed when large amounts of Gd are concentrated within the small intracellular space. Atypical *T*_1_ agent platforms such as free MnCl_2_,^30,31^ or Mn-nanoparticles^32,33^ were explored for cellular MRI. In the +2 oxidation state, Mn^II^-based *T*_1_ agents including nanoparticles typically show a decrease of *T*_1_ relaxivity with increase of magnetic fields above 0.1 T,^34,35^ and are accompanied by relatively strong *T*_2_ effects (darkening) that compromise the *T*_1_ enhancement.^30,36^ In addition, Mn^II^-complexes are kinetically labile in aqueous solution. Despite the above mentioned promising results, highly sensitive, stable and biocompatible *T*_1_ CAs with high cell permeability and retention remains an unmet challenge. To expand this repertoire we decided to explore a different platform to develop *T*_1_ CAs for cellular MRI. Herein we report the design, synthesis, characterization and cell labeling studies of a novel cell-permeable and esterase-activatable *T*_1_ agent based on a Mn^III^-porphyrin (MnP).

## Results and discussion

### Molecular design and principle of strategy

The molecular design started from Mn^III^tetra(carboxy-porphyrin), (MnTCP, 3), a novel Gd-free ECF *T*_1_ agent we developed.^37^ Optimized from the leading compound, Mn^III^tetra(4-sulfonatophenyl)porphyrin (MnTPPS),^38^ MnTCP is the smallest water-soluble MnP synthesized to date.^37^ It displays a fast *in vivo* clearance through renal filtration^39^ and rapid extravasation ideal for cancer detection by dynamic contrast enhanced (DCE) MRI.^40^ Despite a smaller size and thus shorter rotational diffusion time (*τ*_R_), MnTCP exhibits an *r*_1_ of 7.9 mM^−1^ s^−1^, comparable to the larger MnTPPS at a high clinical field of 3 T. This is about twice as sensitive as typical small GBCAs of similar size. To the best of our knowledge, MnTCP has the highest *r*_1_ at clinical magnetic field strengths (1–3 T) among all known small *T*_1_ CAs with molecular weight below 600 dalton. Unlike Mn^II^-based *T*_1_ agents, Mn^III^TCP exhibits much lower negative *T*_2_ effect (*r*_2_/*r*_1_ = 1.15 at 3 T). The rigidity of the porphyrin macrocycle results in a pre-organized metal binding pocket compatible for Mn^III^. This leads to a MnP complex with high thermodynamic and kinetic stability, reducing the likelihood of metal leakage.^41,42^ By eliminating four phenyl rings from MnTPPS, the design of MnTCP minimized the hydrophobic surface areas, with four water-solubilizing carboxylates directly attached at the *meso*-positions of the porphyrin to increase polarity. The reduced lipophilicity together with condensed negatively charged groups helps to avoid interference with cellular molecular machinery, minimizing intracellular compartmentalization,^43^ and also helps to retain MnTCP by preventing leakage through the intact cell membrane, a property well-documented for polyanionic fluorescent cell tracers.^43^ These characteristics of MnTCP make it an ideal precursor to design a cell labeling *T*_1_ agent, if intracellular delivery can been realized.

**Scheme 1:**
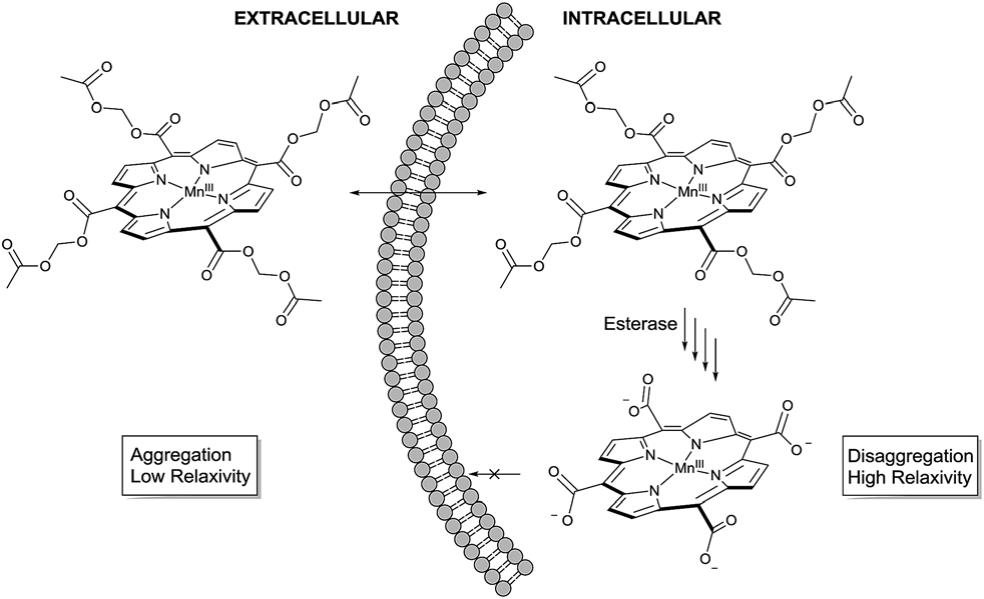
Proposed mechanism for cell uptake and retention.

To noninvasively deliver MnTCP into the cells, our strategy is to mask the polarity of MnTCP by converting polar carboxylate groups to lipophilic AM esters, a prodrug approach well-established for loading otherwise impermeable carboxylate-bearing therapeutics or imaging agents into cells.^44–46^ Unlike most Gd-chelates, of which the carboxylate groups are directly involved in Gd-binding, the four peripheral carboxylate groups in MnTCP are free and available for derivatization with little effect on Mn-affinity. As illustrated in Scheme 1, a permeable AM ester derivative of MnTCP (MnAMP) was designed for crossing the cell membrane.

The lipophilic nature and positively charged Mn^III^-porphyrin core of MnAMP will facilitate the cell uptake. Furthermore, the hydrophobic MnAMP is expected to aggregate in aqueous solution resulting in a state of low relaxivity, a well-characterized phenomenon for a series of known hydrophobic MnPs.^47^ Hydrolysis of AM esters catalyzed by intracellular esterase releases the negatively charged carboxylate that is expected to help disaggregation, thereby inducing increases in relaxivity and facilitating the retention of the agent inside the cell.

### Synthesis and characterizations of MnAMP

**Scheme 2:**
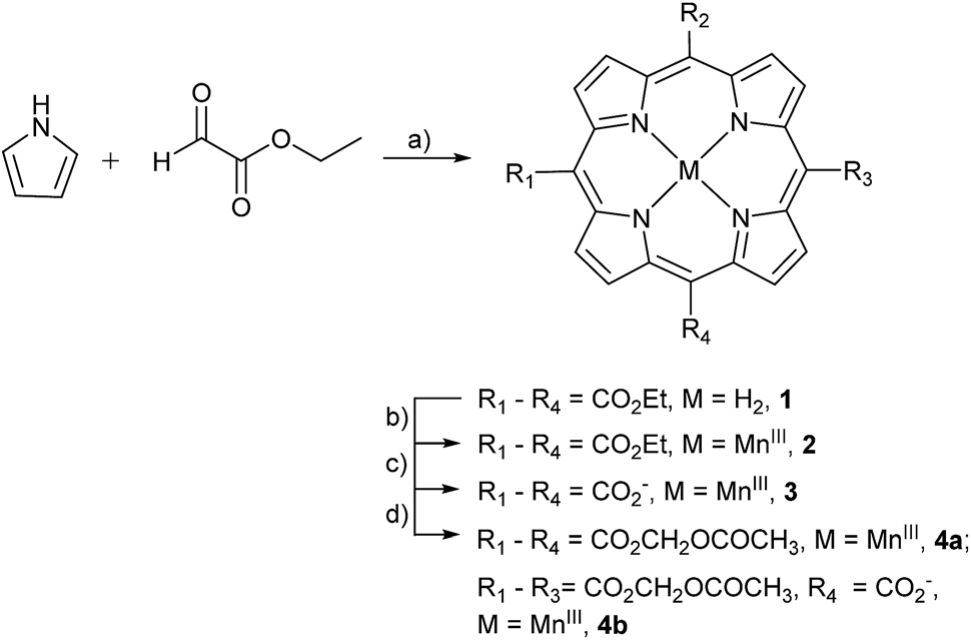
Synthesis of AM derivative of MnTCP. Reagents and conditions: (a) BF_3_OEt_2_, DCM, 25 °C, 10%; (b) MnCl_2_·4H_2_O, DMF, reflux, 85%; (c) NaOH/EtOH/THF, reflux, 85%; (d) DBU, AMBr, DMF, 55 °C, 65%.

The stepwise total synthesis of MnAMP is summarized in Scheme 2. The precursor MnTCP, 3 was synthesized through the hydrolysis of 5,10,15,20-tetra(ethoxycarbonyl)porphyrinato manganese(iii), 2 (ref. 48) under basic conditions. Since the resulted sodium salt of MnTCP is highly polar it is not soluble in DMF. Protonation of the carboxylate groups with 1.0 M HCl was necessary to impart sufficient solubility in DMF for the subsequent installation of the AM ester groups.

Synthesis of MnTAMP, 4a was accomplished with acetoxymethyl bromide (AMBr) and an organic base, diazabicyclo[5.4.0]undec-7-ene (DBU) in DMF at 55 °C for 30 h. During the purification, 4a was shown to be unstable. It decomposes on silica gel during flash chromatography, to form a tris-AM ester derivative (MnTriAMP) 4b with very similar retention time as 4a. Thus, MnTAMP and MnTriAMP were isolated together in 65% yield with a ratio of about 53 : 47 as determined by HPLC-ESI MS (Fig. S5†). Interestingly, the mono-hydrolyzed product 4b is much more stable than 4a with negligible decomposition observed on silica gel and was therefore isolated in a pure form for characterization. We hypothesized that the low stability of 4a may involve the net positive charge on the molecule due to Mn^III^, facilitating the nucleophilic attack of negatively charged hydroxide on the ester carbonyl carbon. The stability of MnTriAMP is owing to the balance of charge between the carboxylate anion and the metal center. To test this hypothesis and to further verify the synthesis, the Mn-free analogue of 4a was prepared starting with apo-porphyrin 1 as a precursor following the same synthetic pathway (Scheme S2†). The following observations are in good agreement that Mn^III^ may activate the single ester hydrolysis: (1) tetra(acetoxymethoxycarbonyl)porphyrin (TAMP), 5, is much more stable than Mn-inserted analogue 4a, since no decomposition was observed during the purification on silica gel; (2) the tetraethyl ester MnP analog, 2, also decomposed slightly during the metal insertion reaction to provide mono-hydrolyzed MnEt_3_P as the only side product,^48^ while Mn-free form 1 is much more stable in ester hydrolysis. The structures of the Mn-free products along this control synthetic pathway were confirmed with NMR, since the paramagnetic Mn^III^ makes it difficult to use NMR for routine structural characterization. All the MnPs were characterized by high resolution ESI-MS, UV-visible and Fourier transform infrared (FTIR) spectroscopy (see ESI†). The purity of the final products was confirmed by HPLC (Fig. S1–S3†) and Mn atomic absorption spectroscopy (AAS).

Since the single negative charge on the carboxylate of MnTriAMP was balanced by Mn^III^ to give an overall neutral compound, MnTriAMP was also expected to cross the cell membrane efficiently. In fact, the similar retention time of 4a and 4b on silica gel TLC as well as reverse phase (C18) HPLC suggest they have similar lipophilicity (Fig. S2†). Upon cellular internalization and esterase hydrolysis, both 4a and 4b would be converted to polar MnTCP, with three net negative charges, thereby trapping the compounds inside the cell. All experiments below were carried out with a mixture of 4a and 4b referred to collectively as MnAMP.

### Low relaxivity of MnAMP due to aggregation

Unlike MnTCP, MnAMP is readily soluble in organic solvents, due to its hydrophobic nature, a property desirable for achieving cell permeability. For the experiments described below, DMSO stock solutions (20 mM) of the hydrophobic MnAMP were infused into the aqueous solution. The expected aggregation behavior of hydrophobic MnAMP in buffer solution was monitored by UV-visible spectroscopy (Fig. S6†), as indicated by gradual decrease of absorption. Dynamic light scattering (DLS) experiment indicated that the average size of the aggregates was ∼450 nm after 2 min in HEPES buffer, further increasing in size to ∼2.4 μm after 2 h (Fig. S7†) without the formation of visible precipitates. The aggregation can be reversed by addition of acetone (Fig. S8†). The significant change, observable on the UV-visible spectrum, makes it possible to monitor the kinetics of aggregation/disaggregation processes optically, as shown in the next section.^47^

**Fig. 1:**
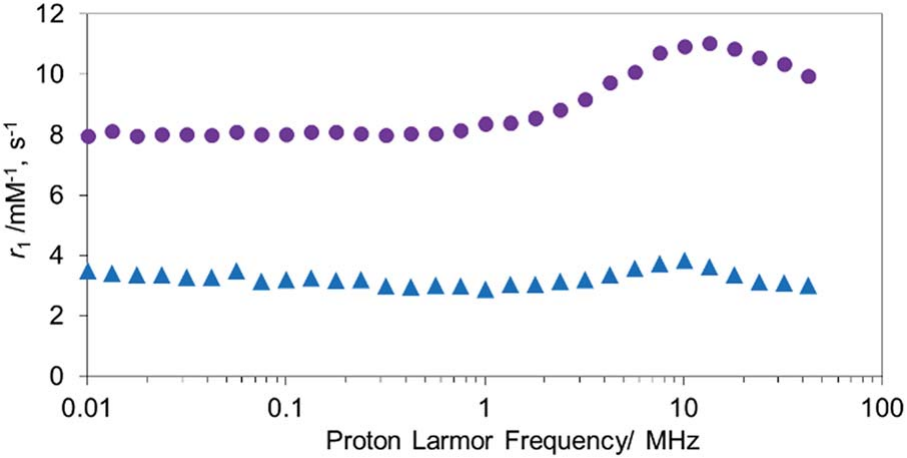
The NMRD profiles of MnTCP (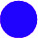), MnAMP (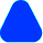) from 0.0001 MHz to 42 MHz at 25 °C.

To confirm the aggregation of MnAMP will lead to a “quenching” state of *r*_1_, the nuclear magnetic relaxation dispersion (NMRD) profile of MnAMP was acquired with a fast field-cycling NMR relaxometer from 0.23 mT up to 1 T, and compared with that of MnTCP^37^ (Fig. 1). Solutions of MnAMP and MnTCP (80 μM in Dulbecco's Modified Eagle Medium, DMEM, 25 °C) were prepared and left in the dark for 2 h to allow for aggregation of MnAMP prior to NMRD measurement. The quantitative *r*_1_ values were normalized to the concentration per paramagnetic metal ion which was calibrated by Mn AAS. As expected, MnAMP exhibits significantly lower relaxivity than MnTCP across the entire magnetic field strength covered by the experiment. At low fields below 1 MHz (0.03 T), MnTCP has an *r*_1_ double that of MnAMP. At the clinically relevant field of 1 T, however, the relaxivity of MnTCP (*r*_1_ = 9.95 mM^−1^ s^−1^) is more than 3-fold higher than that of MnAMP (*r*_1_ = 3.01 mM^−1^ s^−1^). This large difference in relaxivity suggests that MnAMP has good potential as an esterase-activatable CA.

### Esterase-catalyzed hydrolysis of MnAMP

**Fig. 2:**
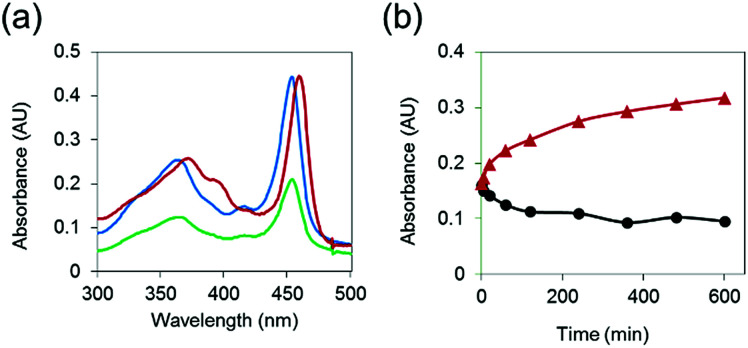
(a) Differences in UV-visible absorbance spectra of MnAMP upon esterase hydrolysis: before esterase addition (blue), 10 h after esterase addition (red), control at 10 h (green) at 25 °C; (b) kinetic trace of esterase hydrolysis (red) and control (black) monitored at 465 nm.

To demonstrate that MnAMP can be hydrolyzed by esterase to lead to disaggregation, partially pre-aggregated MnAMP (60 μM, HEPES buffer) was incubated with porcine liver esterase (3.1.1.1, 3 U mL^−1^), and the reaction was monitored by UV-visible spectroscopy. A gradual increase in absorption and red-shifting of the Soret band (Fig. 2a) could be continuously monitored during the incubation, as opposed to the optical response during the above-mentioned aggregation process. In the control sample without addition of esterase, the absorption continued to slowly decrease over time (Fig. 2b). These results support that esterase hydrolysis leads to disaggregation of MnAMP.

To further confirm that the enzyme-induced disaggregation was due to the stepwise AM ester hydrolysis, the partially-hydrolyzed sample was analyzed by HPLC-MS. Along with the residual MnTriAMP, the expected intermediates, including regio-isomers of MnBiAMP (R_1_, R_2_ = CO_2_^−^, R_3_, R_4_ = CO_2_CH_2_OCOCH_3_ and R_1_, R_3_ = CO_2_^−^, R_2_, R_4_ = CO_2_CH_2_OCOCH_3_), MnMAMP (R_1_–R_3_ = CO_2_^−^, R_4_ = CO_2_CH_2_OCOCH_3_) and product MnTCP (R_1_–R_4_ = CO_2_^−^), were all detected as separated HPLC peaks (Fig. S4†) that were confirmed by ESI-MS (Table S4†). Their order of retention times on a reverse-phase (C18) column is consistent with their relative polarities. Those species were also intermediates for the synthetic step of installing AM esters on MnTCP (Scheme 2), which were detectable by HPLC-MS during the reaction. We noticed, however, that the enzymatic reaction by the isolated esterase does not proceed to the completed hydrolysis. This is not surprising due to the limited scope of substrate diversity and compromised reactivity of isolated enzymes in buffer. Because there are a variety of intracellular esterases^49^ that are more active in live cells than as isolated enzymes, intracellular hydrolysis should proceed to MnTCP more efficiently under cell labeling conditions. This has been repeatedly demonstrated with a wide range of AM ester prodrugs^49,50^ and fluorescent tracers.^46^ As a large family of enzymes, esterases are ubiquitous in mammals and are found in all kingdoms with broad substrate specificity. There are also a variety of esterases present in the cytosol. This led to the development of an assortment of commercially available cell viability and membrane integrity probes utilizing fluorescent sensors with AM esters masking polar carboxylates that are trapped in the cytosol upon esterase hydrolysis.^51,52^

### MRI activation of MnAMP by enzymatic hydrolysis

**Fig. 3:**
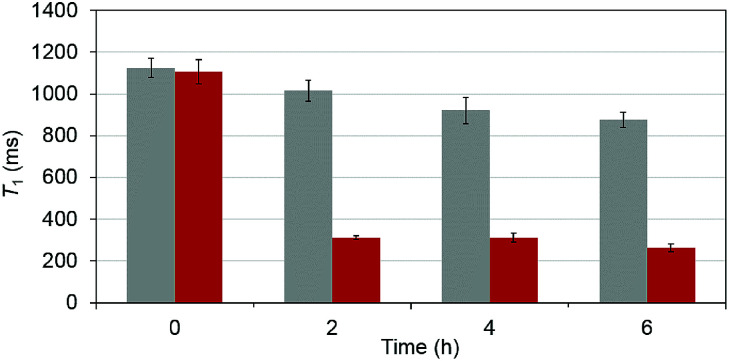
Time-dependent *T*_1_ (ms) of MnAMP solutions in the presence (red) and absence (grey) of porcine liver esterase. All measurements done at 1.5 T, 25 °C, by means of standard inversion recovery techniques. *T*_1,d_ = 2.568 ± 0.017 s, (PBS), fitted by non-linear regression with standard deviation (SD) from the fitting included as error bars.

To test that the esterase catalyzed hydrolysis induces MRI activation of MnAMP, the relaxivity (25 °C, 1.5 T) was measured in the presence and absence of porcine liver esterase (20 U mL^−1^). Solutions of MnAMP (1 mM) in HEPES buffer (50 mM, pH 7.4) were prepared and allowed to pre-aggregate in the dark for 30 min. The *T*_1_ relaxation times were measured prior to addition of esterase and after 2, 4, and 6 h incubation with esterase at 37 °C (Fig. 3).

A significant relaxation enhancement from *T*_1_ = 1.06 s to *T*_1_ = 312 ms was observed after 2 h incubation with esterase. Continuous increase of *T*_1_ relaxivity at a slower rate could be monitored at 4 and 6 h. These results confirmed that MRI activation occurred upon the stepwise hydrolysis of MnAMP. The release of more polar carboxylates broke up the aggregation and increased the water accessibility to the paramagnetic Mn center. Under the current experimental condition, enzymatic activation produced a 3.5-fold increase in *T*_1_ relaxation. Other MRI enzyme sensing strategies have been developed^53^ such as modulation of water coordination number (*q*),^54^ tumbling rate (*τ*_R_),^55^ chemical exchange saturation transfer^56^ and precipitation enhanced staining.^57^

### Biocompatibility of MnAMP

To ensure that MnAMP is biocompatible for cell labeling purposes, we evaluated the toxicity of MnAMP in mammalian cells. A human glioma cell line, U373, was first chosen for the safety test. The extracellular fluid precursor MnTCP was used as a membrane-impermeable control. After incubation with 80 μM MnPs for 2 h, cell proliferation was indirectly examined with an MTT assay 24 and 48 h after labeling. Similar to the untreated cells, proliferation remained unaffected for cells incubated with MnTCP and MnAMP (Fig. S11†). In addition, cell viability > 96% was determined by trypan blue exclusion tests for all three samples, showing good biocompatibility for both MnTCP and MnAMP directly after MnPs incubation, and after 5 h extra growth in fresh medium. Similarly, both MnAMP and MnTCP did not show toxicity effects on MDA-MB-231 cells, a human breast cancer cell line (Table S7†).

### Cell labeling with MnAMP

To test cell permeability and labeling efficiency, approximately 9 × 10^6^ U373 cells were incubated with 80 μM MnAMP or MnTCP for 2 h in growth medium (DMEM), washed 3 times with Hanks Balanced Salt Solution (HBSS), detached and pelleted for MRI and relaxivity measurements. To further examine cell retention of MnAMP, an additional experiment was conducted where MnAMP labeled cells were incubated for a further 5 h in fresh medium in the absence of MnPs.

**Fig. 4:**
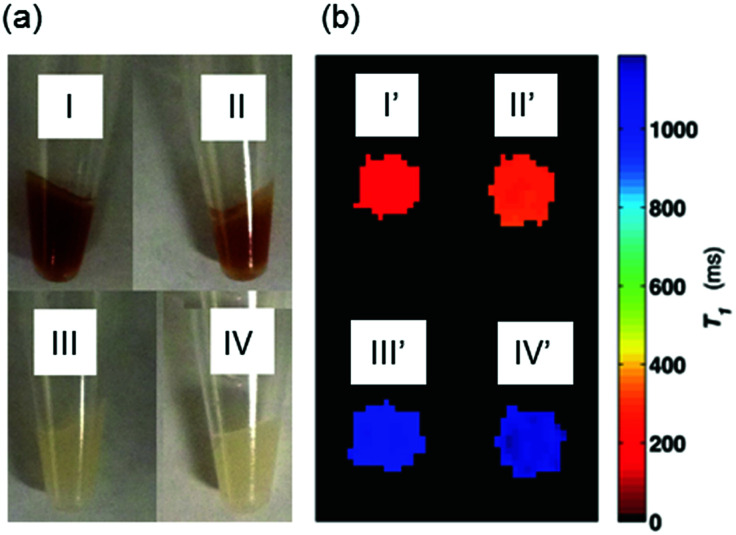
(a) Photograph of cell pellets: (I) MnAMP (II) MnAMP with 5 h in fresh media (III) MnTCP (IV) unlabeled; (b) *T*_1_ maps of the cell pellets estimated from IR-FSE MR images: (I′) MnAMP (II′) MnAMP with 5 h in fresh media (III′) MnTCP (IV′) unlabeled, done at 3 T, room temperature.

As shown in the photograph of the cell pellets (Fig. 4a), the cell uptake of the dark red MnP was clearly visible by eye in cells treated with MnAMP (I) as well as the MnAMP treated sample after 5 h extra growth in fresh medium (II). By comparison, cells treated with MnTCP, which has a similar color as MnAMP, did not show significant color staining (III), similar to the untreated cells (IV), suggesting little cell uptake of MnTCP. Subsequent MRI of the cell pellets was conducted on a 3 T MRI scanner using a *T*_1_-weighted inversion recovery fast spin-echo pulse sequence. As shown in Fig. 4b, the MR image (*T*_1_ map) exhibited significant positive contrast enhancement for MnAMP treated cells (I′), with enhancement maintained after 5 h extra growth (II′), in comparison to unlabeled cells (IV′) and MnTCP-treated control (III′). The relaxation times, *T*_1_ and *T*_2_, of the cell pellets were calculated either from the MR images (at 3 T) or by relaxometry (*T*_1_ at 1 T). As summarized in Table 1, the untreated cells exhibit *T*_1_ and *T*_2_ similar to typical literature values.^58^ A significantly shorter *T*_1_ of 161 ± 4 ms was determined at 3 T for cells treated with MnAMP. The 5 h retention sample resulted in a slight increase of *T*_1_ to 272 ± 12 ms but still maintained the majority of *T*_1_ enhancement, compared to 1134 ± 18 ms for the untreated cells. Cellular *T*_1_ enhancement and retention of MnAMP was also confirmed by relaxivity measurement at 1 T. Notice that the systematically shorter *T*_1_ values for all samples at 1 T compared to those measured at 3 T are consistent with the relaxation dispersion behaviors of MnTCP and unlabeled tissues. Even though *T*_2_ enhancement was detectable for MnAMP treated cells (45% *T*_2_ shorting), the *T*_1_ effect is dominant. This is significantly different from Mn^II^-based CAs, which show strong *T*_2_ effects (darkening) that compromise the positive *T*_1_ enhancement.^30^ By comparison, MnTCP treated cells exhibited a negligible decrease in *T*_1_ (at 1 T and 3 T) or *T*_2_ (at 3 T), thereby confirming that MnTCP is cell-impermeable and its permeability was dramatically enhanced through addition of the lipophilic AM ester groups.

**Table 1:** *T*_1_, *T*_2_ and Mn content of U373 cells labeled with MnAMP, MnAMP after 5 h in medium and MnTCP

Sample	*T*_1_ (ms), 1 T^a^	*T*_1_ (ms), 3 T^b^	*T*_2_ (ms), 3 T^b^	Mn/cell (moles)^d^
Control	969 ± 69	1134 ± 18	101 ± 26	N/A
MnAMP	95 ± 11	161 ± 4	44 ± 2	2.804 × 10^−15^ ± 0.093 × 10^−15^
MnAMP 5h^c^	147 ± 17	272 ± 12	55 ± 6	1.536 × 10^−15^ ± 0.390 × 10^−15^
MnTCP	879 ± 10	1048 ± 15	107 ± 48	4.280 × 10^−17^ ± 0.093 × 10^−17^

a Data at 1 T was acquired by relaxometry by means of standard inversion recovery techniques, fitted by non-linear regression with SD from the fitting.

b Data at 3 T was quantified from the MR images based on a pixel-by-pixel relaxation time analysis, the SD represents the variation among the pixels.

c Cells labeled with MnAMP were grown in MnP-free medium for extra 5 h.

d Data are shown as means ± SD of three independent experiments.

With a relatively low incubation concentration (80 μM) and short incubation time (2 h), the observed significant *T*_1_ enhancement of labeled cells demonstrated that MnAMP has high labeling efficiency (Table 1). In comparison, the known small molecule *T*_1_ agents often require higher concentrations and longer incubation times, up to 24 h, to achieve sufficient *T*_1_ enhancement.^59^ The most efficient cell labeling *T*_1_ agent reported to date is a Gd-loaded glucan particle (Gd-GP). Macrophages incubated with 250 μM of Gd-GP for 24 h resulted in an *R*_1_ = 3.6 s^−1^ (*T*_1_ = 278 ms) at 1 T.^60^ In contrast, with lower concentration and shorter incubation time, MnAMP labeled cells reached even higher *T*_1_ enhancement with an *R*_1_ = 10.6 s^−1^ (95 ms) at 1 T. To the best of our knowledge MnAMP is the most efficient *T*_1_ agent developed for cell labeling to date.

To confirm whether MnAMP is converted to MnTCP intracellularly, the cells were lysed and the cytosolic and membrane fractions were isolated and analyzed by HPLC and Mn-AAS, respectively. As expected all of the MnAMP was completely converted to MnTCP (Fig. S14–S17†) and was mainly found in the cytosol. In contrast, no MnP was detected in the membrane fraction.

### Quantitative cell uptake of MnAMP

For quantitative analysis of cell uptake, all the cell pellets were digested with 60% nitric acid in an ultrasonic bath at 40 °C for 7 h to obtain homogenous solutions. The cell digests were submitted for further Mn-quantification by graphite furnace Mn-AAS. Data reported on a per cell basis (Table 1). Unlike Gd, Mn is an essential micronutrient present in cells. The endogenous intracellular [Mn] was subtracted as background signal from all calculations. An increase in cellular Mn content 2.8 × 10^−15^ mole per cell was determined directly after labeling, corresponding to 1.7 × 10^9^ Mn atoms per cell. Since the uptake of paramagnetic metal ion per cell is within the range of recently developed *T*_1_ cell labeling agents,^27,60,61^ the high cellular contrast enhancement likely results from the combination of high cell uptake together with higher *T*_1_ relaxivity of MnPs at high fields compared to small molecule GBCAs and Gd-nanoparticles.^62^ MnAMP also demonstrated good retention after 5 h in fresh medium with only a slight decrease in *T*_1_ enhancement.

In comparison the increase of Mn content for the cell-impermeable control MnTCP was minimal with only 4.3 × 10^−17^ mole per cell as expected from the lack of contrast enhancement. These results demonstrate that MnAMP is one of the most efficient *T*_1_ cell labeling agents to date.^60^

### Labeling of a different cell line with MnAMP

To demonstrate that MnAMP can be applied to label different cell types, a breast cancer cell line, MDA-MB-231, was chosen for the comparative labeling study. The same conditions used for the U373 cell line was applied to label MDA-MB-231 cells with MnAMP (80 μM, 2 h). A significant contrast enhancement with a *T*_1_ of 215 ± 12 ms was determined for the cell pellet labeled with MnAMP, compared to 1072 ± 28 ms for the control sample treated with MnTCP and a *T*_1_ of 1107 ± 27 ms for untreated cells. These values are comparable to the results obtained with the U373 cell line described above. Similarly, neither MnAMP nor MnTCP was toxic to MDA-MB-231 cells, as determined *via* a trypan blue viability assay. The MDA-MB-231 cell line had been previously examined for MRI labeling with MnCl_2_ at several concentrations, and had shown relatively strong uptake of MnCl_2_.^63^ Notably, a *T*_1_ of 320 ms was obtained for MnCl_2_ labeled cells, which is substantially larger than the *T*_1_ for MnAMP labeled MDA-MB-231 cells despite a higher incubation concentration (200 μM MnCl_2_).^31^ Furthermore the *T*_2_ effect is significantly stronger for MnCl_2_ compared to MnAMP, compromising the *T*_1_ efficiency.

Many previous studies demonstrated successful MRI cell labeling on phagocytes, such as macrophages and dendritic cells. These phagocytes can take up particulate MRI CAs through phagocytosis, even though the labeling agents have limited cell permeability. A variety of approaches have been developed to label non-phagocytic cells^64^ such as the attachment of cell penetrating peptides,^23–26^ functionalization of nanoparticles to promote receptor mediated endocytosis,^65^ or by physical perturbation of the cell membrane.^66^ The two cell lines used in the current study are not considered as typical highly phagocytic cell types. The high level of cell uptake of MnAMP is thus likely due to high permeability. Therefore, MnAMP is a safe and efficient *T*_1_ agent and can potentially be applied for labeling a variety of different cell types.

## Experimental

### Chemicals and reagents

For synthetic procedures all reagents and solvents were of commercial reagent grade and were used without further purification except where noted. Reagents were purchased from Sigma-Aldrich. Solvents were purchased from Caledon Labs. All reactions were carried out with oven dried glassware, anhydrous solvents and under argon atmosphere unless stated otherwise. Biotechnology grade (4-(2-hydroxyethyl)-1-piperazineethanesulfonic acid) (HEPES) buffer (typically used as 10 mM solution, pH = 7.4, ionic strength = 100 mM, except where noted), Hanks Balanced Salt Solution (HBSS) was purchased from Life Technologies and ammonium acetate was purchased from Fisher Scientific. Phosphate buffer saline was purchased from Sigma Life Science, sterile filtered and endotoxin tested. Ultrapure water was generated by a MilliQ system. Porcine liver esterase (3.1.1.1.) was purchased from Sigma-Aldrich (E3019) as a lyophilized powder, 17 U mg^−1^. The MDA-MB-231 cell line was obtained from ATCC (American Tissue Culture Collection Manassas, VA, USA). The human glioma cell line U373 was gifted to us by Dr Janusz Rak. Trypsin EDTA was purchased from Gibco (Carlsbad, CA, USA).

### Synthesis of MnAMP

Protonated MnTCP^37^ (55.6 mg, 103 μmol) was suspended in DMF (8 mL). Under continuous stirring, DBU (80 μL, 535 μmol, 5 eq.) was added dropwise. AMBr (130 μL, 1.3 mmol, 13 eq.) was added in three separated portions at 10 min, 6 h and 24 h and the progress of the reaction was monitored by TLC. The reaction temperature was maintained at 55 °C for 30 h. Distillation of DMF under reduced pressure resulted in a crude dark oil. DCM was added to the crude material to dissolve the product and the mixture was filtered. The DCM layer was neutralized with water and brine twice each. The organic layer was dried over sodium sulfate and filtered prior to concentration on a rotary evaporator. Purification by flash column chromatography (eluting with 5% to 10% MeOH in DCM) on silica gel gave 55.4 mg (65%) of 4a and 4b (53 : 47 ratio by HPLC-MS) as a red-brown solid. Due to decomposition of 4a to 4b on silica gel it was not possible to isolate 4a. The purest fraction that contained 4a was further isolated by preparative TLC and was analyzed by LCMS and gave a ratio of 4a : 4b (83 : 17). Pure 4b was isolated from the mixture by flash chromatography, with the same elution solvents, 2 more times. Characterization of 4a: *R*_f_ = 0.27 (10% MeOH in DCM), ESI MS positive mode: found *m*/*z* = 827.0885 [M^+^], calcd for C_36_H_28_MnN_4_O_16_^+^, *m*/*z* = 827.0875, and characterization of 4b: *R*_f_ = 0.33 (10% MeOH in DCM), UV-Vis (HEPES buffer): *λ*_abs_ = 376, 395, 416, 457, 556 nm, *λ*_max_ = 457 nm; IR (neat): *ν* = 2921, 2852 (sp^3^ C–H, methyl alkane), 1743 (sp^2^ C=O, carbonyl), 1553 (sp^2^ COO^−^), 1192 (C–O single bond, ester) cm^−1^; ESI MS found *m*/*z* = 755.0678 ([M]^+^), calcd for C_33_H_24_MnN_4_O_14_^+^, *m*/*z* = 755.0664.

### UV-visible spectroscopy

UV-visible spectra were recorded on an Agilent 8453 UV-visible spectroscopy system. The extinction coefficient (*ε*) of 3 was previously reported.^37^ Absorption spectra of 3, 4b, MnAMP, and 5 were measured in HEPES buffer at 25 °C.

### Relaxivity measurements

The NMRD profiles were acquired with a fast field-cycling NMR relaxometer (SpinMaster FFC2000 1T C/DC, Stelar, s.r.l.) at Imaging Research Laboratories, Robarts Research Institute, Western University, Ontario. All NMRD experiments were acquired with a controlled temperature of 25 °C, at 30 individual relaxation fields, logarithmically distributed from 0.01 to 42.485 MHz and using an acquisition field of 16.2 MHz. MnTCP was dissolved in ultrapure water and MnAMP was dissolved in DMSO to provide 20 mM stock solutions that were infused into DMEM for NMRD measurements at a final concentration of 80 μM (quantified by AAS), with 0.5% DMSO at 25 °C. The samples were allowed to aggregate for 2 h in the dark prior to NMRD measurement.

### Esterase hydrolysis of MnAMP monitored by UV-visible spectroscopy

Esterase hydrolysis was carried out with MnAMP (final concentration: 60 μM, 0.5% DMSO) in HEPES buffer at 25 °C, with porcine liver esterase (3.1.1.1) at 3 units per mL (U mL^−1^) activity. Prior to addition of esterase, the solution of MnAMP was left to aggregate for 30 min. The absorbance was monitored using a 1 mm quartz cuvette. Upon addition of esterase the reaction and control were placed on a mechanical shaker (400 rpm). The absorption spectra for both control and esterase reaction were monitored for 12 h. The experiment was duplicated.

### Esterase hydrolysis monitored by relaxivity

The observed longitudinal water proton relaxation times (*T*_1,obs_) were measured on a HTS-100 Cryomagnet coupled with a fast field-cycling NMR relaxometer (SMARTracer, Stelar, s.r.l.) operating at 1.5 T (64 MHz), by means of the standard inversion-recovery technique (16 experiments, 2 scans). Esterase hydrolysis was carried out with MnAMP (final concentration: 1 mM) in 50 mM HEPES buffer (ionic strength = 0.5 M, pH = 7.4) at 37 °C, with porcine liver esterase (3.1.1.1) at 20 U mL^−1^ activity. The substrate was allowed to aggregate for 30 min before addition of the enzyme. Relaxivities were recorded prior to and following incubation at 37 °C for 2, 4 and 6 h. The experiment was duplicated.

### Cell labeling, viability and MR analysis

The human glioma cell line U373 was grown at 37 °C with 5% CO_2_ in DMEM supplemented with 10% fetal bovine serum and 1% penicillin/streptomycin (all components from Life Technologies Inc., Burlington, ON, Canada). CAs were quantified for manganese content by AAS prior to cell labeling. MnTCP was dissolved in ultrapure water and MnAMP was dissolved in DMSO to give 20 mM stock solutions. The stock solutions were added to the medium with the cells resulting in 80 μM (0.5% DMSO) final concentrations for incubation of MnTCP or MnAMP for 2 h. The cells were washed with HBSS to remove the extracellular CA, and were either harvested directly, or after 5 h additional growth in fresh medium without MnPs, to test the retention of intracellular MnPs. Trypsin EDTA (0.25%) was added to detach the cells. Cells were counted and viability was assessed by trypan blue exclusion assay. In addition, cell proliferation was also assessed with an MTT assay according to manufacturer's instructions (Vybrant MTT Cell Proliferation Assay Kit, Life Technologies Inc., Burlington, ON, Canada). The cells were pelleted for MRI (Fig. S12 and S13†) and relaxivity measurement.

Cellular MR images were acquired on a Discovery MR750 3.0 T clinical scanner (General Electric Healthcare, Waukesha, WI U.S.A.) using an insertable gradient coil (inner diameter = 17.5 cm, gradient strength = 500 mT m^−1^, and peak slew rate = 3000 T m^−1^ s^−1^), and a solenoid radiofrequency coil (inner diameter = 1.5 cm), both of which were custom-built at Robarts Research Institute. A *T*_1_-weighted inversion recovery fast spin-echo pulse sequence was used with inversion times (TI) of 50, 75, 100, 150, 250, 350, 500, 750, 1000, 1250, 1500, 2000, and 2500 ms and Repetition Time (TR) = 3000 ms, Echo Time (TE) = 18.5 ms, Echo Train Length (ETL) = 2, 1 mm slice thickness (THK), FOV = 40 × 40 mm, matrix size of 128 × 128 points, and receive bandwidth (BWr) of 15.63 kHz. For the spin–spin relaxation time (*T*_2_) estimations a spin echo pulse sequence was used with TE = 20, 30, 40, 50, 60, 70, 80, 90, and 100 ms, TR = 600 ms, THK = 0.3 mm, BWr = 20.83 kHz, with a matrix of 128 × 128 points, FOV = 30 × 30 mm, and 4 averages. Zero filling interpolation was applied to the spin-echo images. The signal intensity was analyzed to give the relaxation times *T*_1_ and *T*_2_ of the cell pellets. Matlab was used to generate non-linear fits for each pixel to curves defined by the following equations:For *T*_1_: *S* = *M*_z_ − (*M*_z_ − *M*_i_)e^−*t*/*T*_1_^For *T*_2_: *S* = *M*_xy_e^−*t*/*T*_2_^ + *n*_off_where *M*_z_ is the steady state longitudinal magnetization at thermal equilibrium, *M*_i_ is the magnitude of the inverted magnetization acquired during the readout, *M*_xy_ is the transverse magnetization, and *n*_off_ is any signal offset present in the images.

### Cell lysis, HPLC and UV-Vis analysis after labeling

After cell labeling and testing of viability, the cells were re-suspended in 500 μL PBS with 0.01% saponin.^67^ After 30 min at 25 °C the cells were centrifuged at 1000*g* for 5 min. The supernatant was collected as the cytosolic fraction. To the pellet was added 500 μL PBS followed by 50 strokes on a Dounce homogenizer and centrifugation at 15 000*g*. The supernatant was collected as the nuclear fraction with the remaining pellet collected as the membrane fraction. The cytosolic fractions were analysed by HPLC and UV-visible spectroscopy (Fig. S14–S18†). The membrane fractions were digested and quantified by GFAAS.

### Graphite furnace atomic absorption spectroscopy

Mn-quantification was determined with a ThermoFisher GFS 35 graphite furnace absorption spectrometer equipped with an electrothermal atomizer, an autosampler and a deuterium-lamp background correction system. A Perkin-Elmer Intensitron manganese hollow-cathode lamp was used according to the manufacturer's recommendations. Table S1† shows the electrothermal program for the determination of manganese. A 10% w/v magnesium nitrate solution (Aldrich, Germany), was used as the chemical modifier. A 1000 mg mL^−1^ manganese (2% HNO_3_) Titrisol (Aldrich) was used for preparation of standard working solutions. Nitric acid Suprapure (Aldrich) was used for stabilization of samples and working standards. Standard solutions for calibration purposes were prepared by proper dilution with 2% w/v HNO_3_ solution. A rinsing step was included prior to withdrawal of each aliquot. Spectroscopic analyses of samples were performed with 15 μL of standard/sample and 5 μL modifier injected sequentially into the graphite furnace atomizer. Measurements were performed in triplicate. The cellular elemental concentration was determined by dividing the total content by the number of cells. The endogenous Mn content determined in the unlabeled cells was subtracted from the MnP labeled cells.

## Conclusions

In summary, we have designed, synthesized and characterized MnAMP, a cell-permeable and esterase-activatable *T*_1_ CA specifically developed for MRI cell labeling. The novel paramagnetic porphyrin, MnAMP is constructed by rational structural modification of MnTCP, a membrane-impermeable CA with high stability and high *T*_1_ relaxivity. Reaction of MnTCP with AMBr under basic condition successfully converted the polar carboxylate groups to lipophilic AM esters to give MnAMP as a mixture of MnTAMP (4a) and MnTriAMP (4b). As a prodrug of MnTCP, MnAMP is cell-permeable and exhibits low extracellular relaxivity due to aggregation in the aqueous medium. We have demonstrated that AM ester groups in MnTAMP can be catalytically hydrolyzed by a commercially available liver esterase to release the polar carboxylates, inducing disaggregation and thereby, significantly increasing *T*_1_ relaxivity. We have further shown that MnAMP can effectively cross the cell membrane and is converted to MnTCP by intracellular esterase, leading to intracellular accumulation. In comparison the cell uptake of negatively charged MnTCP was negligible, confirming that installation of the AM ester groups was necessary to enhance cell permeability. Highly efficient MRI labeling of two types of non-phagocytic human cells, including a glioma cell line and a breast cancer cell line, was achieved with a relatively low concentration (80 μM) of MnAMP and a short incubation time (2 h). Unprecedentedly strong *T*_1_ enhancement of labeled U373 cells was determined at clinical fields of 1 T (*T*_1_ = 95 ms) and 3 T (*T*_1_ = 161 ms), corresponding to about 10-fold and 7-fold *T*_1_ shortening at both field strengths, respectively. In contrast, the negative *T*_2_ effect was much less significant, suggesting MnAMP primarily acts as a positive agent. Cell viability and proliferation remained unaffected by MnAMP. Therefore, MnAMP is biocompatible and to the best of our knowledge, is the most efficient *T*_1_ cell labeling CA available to date. The current work has demonstrated the potential of MnAMP to be widely applied to label different cell types for *in vivo* monitoring at the commonly used high clinical field of 3 T. Future studies will be focused on *in vivo* applications involving therapeutic cells, including stem cells and dendritic cells for monitoring and optimization of adoptive immunotherapy and stem cell transplantation.

## Supplementary Material

SC-007-C5SC04252F-s001

## References

[cit1] Restifo N. P., Dudley M. E., Rosenberg S. A. (2012). Nat. Rev. Immunol..

[cit2] Rafii S., Lyden D. (2003). Nat. Med..

[cit3] Björklund L. M., Sánchez-Pernaute R., Chung S., Andersson T., Chen I. Y. C., McNaught K. S. P., Brownell A.-L., Jenkins B. G., Wahlestedt C., Kim K.-S., Isacson O. (2002). Proc. Natl. Acad. Sci. U. S. A..

[cit4] Schroeder T. (2008). Nature.

[cit5] Aarntzen E. H., Srinivas M., Walczak P., Janowski M., Heerschap A., de Vries I. J., Figdor C. G., Bulte J. W., Oyen W. J. (2012). J. Nucl. Med..

[cit6] Ahrens E. T., Bulte J. W. M. (2013). Nat. Rev. Immunol..

[cit7] Janjic J. M., Ahrens E. T. (2009). Wiley Interdiscip. Rev.: Nanomed. Nanobiotechnol..

[cit8] Ahrens E. T., Flores R., Xu H., Morel P. A. (2005). Nat. Biotechnol..

[cit9] Tirotta I., Mastropietro A., Cordiglieri C., Gazzera L., Baggi F., Baselli G., Bruzzone M. G., Zucca I., Cavallo G., Terraneo G., Baldelli Bombelli F., Metrangolo P., Resnati G. (2014). J. Am. Chem. Soc..

[cit10] Heyn C., Ronald J. A., Mackenzie L. T., MacDonald I. C., Chambers A. F., Rutt B. K., Foster P. J. (2006). Magn. Reson. Med..

[cit11] Shapiro E. M., Skrtic S., Sharer K., Hill J. M., Dunbar C. E., Koretsky A. P. (2004). Proc. Natl. Acad. Sci. U. S. A..

[cit12] Bulte J. W. M. (2009). AJR, Am. J. Roentgenol..

[cit13] Schenck J. F. (1996). Med. Phys..

[cit14] Muja N., Bulte J. W. M. (2009). Prog. Nucl. Magn. Reson. Spectrosc..

[cit15] Caravan P., Ellison J. J., McMurry T. J., Lauffer R. B. (1999). Chem. Rev..

[cit16] Crich S. G., Biancone L., Cantaluppi V., Duò D., Esposito G., Russo S., Camussi G., Aime S. (2004). Magn. Reson. Med..

[cit17] Aime S., Castelli D. D., Crich S. G., Gianolio E., Terreno E. (2009). Acc. Chem. Res..

[cit18] Guenoun J., Koning G. A., Doeswijk G., Bosman L., Wielopolski P. A., Krestin G. P., Bernsen M. R. (2012). Cell Transplant..

[cit19] Bruckman M. A., Hern S., Jiang K., Flask C. A., Yu X., Steinmetz N. F. (2013). J. Mater. Chem. B.

[cit20] Hooker J. M., Datta A., Botta M., Raymond K. N., Francis M. B. (2007). Nano Lett..

[cit21] Geninatti Crich S., Bussolati B., Tei L., Grange C., Esposito G., Lanzardo S., Camussi G., Aime S. (2006). Cancer Res..

[cit22] Figueiredo S., Cutrin J. C., Rizzitelli S., De Luca E., Moreira J. N., Geraldes C. F., Aime S., Terreno E. (2013). Mol. Imag Biol..

[cit23] Bhorade R., Weissleder R., Nakakoshi T., Moore A., Tung C.-H. (2000). Bioconjugate Chem..

[cit24] Allen M. J., Meade T. J. (2003). JBIC, J. Biol. Inorg. Chem..

[cit25] Olson E. S., Jiang T., Aguilera T. A., Nguyen Q. T., Ellies L. G., Scadeng M., Tsien R. Y. (2010). Proc. Natl. Acad. Sci. U. S. A..

[cit26] Endres P. J., MacRenaris K. W., Vogt S., Meade T. J. (2008). Bioconjugate Chem..

[cit27] Carney C. E., MacRenaris K. W., Mastarone D. J., Kasjanski D. R., Hung A. H., Meade T. J. (2014). Bioconjugate Chem..

[cit28] Yamane T., Hanaoka K., Muramatsu Y., Tamura K., Adachi Y., Miyashita Y., Hirata Y., Nagano T. (2011). Bioconjugate Chem..

[cit29] Kim T., Momin E., Choi J., Yuan K., Zaidi H., Kim J., Park M., Lee N., McMahon M. T., Quinones-Hinojosa A., Bulte J. W. M., Hyeon T., Gilad A. A. (2011). J. Am. Chem. Soc..

[cit30] Aoki I., Takahashi Y., Chuang K.-H., Silva A. C., Igarashi T., Tanaka C., Childs R. W., Koretsky A. P. (2006). NMR Biomed..

[cit31] Nofiele J. T., Cheng H.-L. M. (2013). PLoS One.

[cit32] Kim T., Momin E., Choi J., Yuan K., Zaidi H., Kim J., Park M., Lee N., McMahon M. T., Quinones-Hinojosa A., Bulte J. W. M., Hyeon T., Gilad A. A. (2011). J. Am. Chem. Soc..

[cit33] Faucher L., Tremblay M., Lagueux J., Gossuin Y., Fortin M.-A. (2012). ACS Appl. Mater. Interfaces.

[cit34] Koenig S. H., Baglin C., Brown 3rd R. D., Brewer C. F. (1984). Magn. Reson. Med..

[cit35] Guillet-Nicolas R., Laprise-Pelletier M., Nair M. M., Chevallier P., Lagueux J., Gossuin Y., Laurent S., Kleitz F., Fortin M.-A. (2013). Nanoscale.

[cit36] Pan D., Caruthers S. D., Senpan A., Schmieder A. H., Wickline S. A., Lanza G. M. (2011). Wiley Interdiscip. Rev.: Nanomed. Nanobiotechnol..

[cit37] Cheng W., Haedicke I. E., Nofiele J., Martinez F., Beera K., Scholl T. J., Cheng H.-L. M., Zhang X.-a. (2014). J. Med. Chem..

[cit38] Koenig S. H., Brown III R. D., Spiller M. (1987). Magn. Reson. Med..

[cit39] Cheng H.-L. M., Haedicke I. E., Cheng W., Nofiele J. T., Zhang X.-a. (2014). J. Magn. Reson. Imaging.

[cit40] Nofiele J. T., Haedicke I. E., Zhu K. Y. L., Zhang X.-a., Cheng H.-L. M. (2015). J. Magn. Reson. Imaging.

[cit41] Hambright P. (1977). J. Inorg. Nucl. Chem..

[cit42] Lyon R. C., Faustino P. J., Cohen J. S., Katz A., Mornex F., Colcher D., Baglin C., Koenig S. H., Hambright P. (1987). Magn. Reson. Med..

[cit43] Flourescent Tracers of Cell Morphology and Fluid Flow, in A Guide to Fluorescent Probes and Labeling Technologies, ed. L. Johnson and M. T. Z. Spence, Life Technologies Corporation, 11^th^ edn, 2010, vol. 14, pp. 607–648

[cit44] Tsien R. Y. (1981). Nature.

[cit45] Jansen A. B. A., Russell T. J. (1965). J. Chem. Soc..

[cit46] Assays for Cell Viability, Proliferation and Function, in A Guide to Fluorescent Probes and Labeling Technologies, ed. L. Johnson and M. T. Z. Spence, Life Technologies Corporation, 11^th^ edn, 2010, vol. 15, pp. 833–873

[cit47] Kellar K. E., Foster N. (1992). Inorg. Chem..

[cit48] Trova M. P., Gauuan P. J. F., Pechulis A. D., Bubb S. M., Bocckino S. B., Crapo J. D., Day B. J. (2003). Bioorg. Med. Chem..

[cit49] Liederer B. M., Borchardt R. T. (2006). J. Pharm. Sci..

[cit50] Rautio J., Kumpulainen H., Heimbach T., Oliyai R., Oh D., Jarvinen T., Savolainen J. (2008). Nat. Rev. Drug Discovery.

[cit51] Papadopoulos N. G., Dedoussis G. V., Spanakos G., Gritzapis A. D., Baxevanis C. N., Papamichail M. (1994). J. Immunol. Methods.

[cit52] Woodroofe C. C., Won A. C., Lippard S. J. (2005). Inorg. Chem..

[cit53] Hingorani D. V., Yoo B., Bernstein A. S., Pagel M. D. (2014). Chem.–Eur. J..

[cit54] Moats R. A., Fraser S. E., Meade T. J. (1997). Angew. Chem., Int. Ed..

[cit55] Rodriguez E., Nilges M., Weissleder R., Chen J. W. (2010). J. Am. Chem. Soc..

[cit56] Chauvin T., Durand P., Bernier M., Meudal H., Doan B. T., Noury F., Badet B., Beloeil J. C., Toth E. (2008). Angew. Chem., Int. Ed..

[cit57] Westmeyer G. G., Emer Y., Lintelmann J., Jasanoff A. (2014). Chem. Biol..

[cit58] Bottomley P. A., Foster T. H., Argersinger R. E., Pfeifer L. M. (1984). Med. Phys..

[cit59] Gianolio E., Stefania R., Di Gregorio E., Aime S. (2012). Eur. J. Inorg. Chem..

[cit60] Figueiredo S., Cutrin J. C., Rizzitelli S., De Luca E., Moreira J. N., Geraldes C. F. G. C., Aime S., Terreno E. (2013). Mol. Imag Biol..

[cit61] Trivedi E. R., Ma Z., Waters E. A., Macrenaris K. W., Subramanian R., Barrett A. G., Meade T. J., Hoffman B. M. (2014). Contrast Media Mol. Imaging.

[cit62] Di Corato R., Gazeau F., Le Visage C., Fayol D., Levitz P., Lux F., Letourneur D., Luciani N., Tillement O., Wilhelm C. (2013). ACS Nano.

[cit63] Nofiele J. T., Czarnota G. J., Cheng H.-L. M. (2014). Mol. Imaging.

[cit64] Methods for Labeling Nonphagocytic Cells with MR Contrast Agents, in Molecular and Cellular MR Imaging, ed. M. M. J. J. Modo and J. W. M. Bulte, CRC Press, 2007, pp. 300–306

[cit65] Ahrens E. T., Feili-Hariri M., Xu H., Genove G., Morel P. A. (2003). Magn. Reson. Med..

[cit66] Di Gregorio E., Ferrauto G., Gianolio E., Aime S. (2013). Contrast Media Mol. Imaging.

[cit67] Jamur M. C., Oliver C. (2010). Methods Mol. Biol..

