# Supplementary material for: An enzyme-activatable and cell-permeable MnIII-porphyrin as a highly efficient T1 MRI contrast agent for cell labeling
Source: Chem Sci. 2016 Mar 16;7(7):4308–17. doi: 10.1039/c5sc04252f (PMC6013825; doi:10.1039/c5sc04252f)
Supplement: SC-007-C5SC04252F-s001 [file SC-007-C5SC04252F-s001.pdf]

Electronic Supplementary Material (ESI) for Chemical Science.
This journal is © The Royal Society of Chemistry 2016

Supporting Information

An Enzyme-Activatable and Cell-Permeable Mn^{III}-Porphyrin as a Highly Efficient T_1 MRI Contrast Agent for Cell Labeling

Inga E. Haedicke, Tan Li, Yong Le K. Zhu, Francisco Martinez, Amanda M. Hamilton, Donna H. Murrell, Joris T. Nofiele, Hai-Ling M. Cheng, Timothy J. Scholl,* Paula J. Foster,* and Xiao-an Zhang**

1. Abbreviations S2
2. Synthesis (Scheme S1-S2) S2-S6
3. Graphite Furnace Atomic Absorption Spectroscopy (Table S1) S7
4. Liquid Chromatography-Mass Spectrometry (Figure S1-S4 & Table S2-S5) S8-S11
5. Aggregation of MnAMP (Figure S6-S8) S12-S13
6. Esterase Hydrolysis (Figure S9-S10 & Table S5) S14-S16
7. Cell Labeling, Viability, MRI (Figure S11-S19 & Table S6-S8) S17-S22
8. Spectra for Structural Characterizations (Figure S20-S25) S23-S25
9. References S26

1. Abbreviations

AAS, atomic absorption spectroscopy; AM, acetoxymethyl ester, AMBr, acetoxymethyl bromide; CA, contrast agent; DCE, dynamic contrast enhanced; DMEM, Dulbecco's modified eagle medium; DBU, diazabicyclo[5.4.0]undec-7-ene; ECF, extracellular fluid; FOV, field of view; GBCA, gadolinium based contrast agent; Gd-GP, gadolinium loaded glucan particle; HBSS, hanks balanced salt solution; HEPES, 4-(2-hydroxyethyl)-1-piperazineethanesulfonic acid; MnP, Mn^{III}-porphyrin; MnTPPS, Mn^{III}-tetra(4-sulfonatophenyl)porphyrin; $MnEt_3P$, Mn^{III}-5-carboxy-10,15,20-tris(ethoxycarbonyl)porphyrin; MRI, magnetic resonance imaging; NMRD, nuclear magnetic relaxation dispersion; τ_R, rotational diffusion time; SPIO, superparamagnetic iron oxide nanoparticle; T, Tesla; T_1, longitudinal proton relaxation time; T_2, transverse proton relaxation time.

2. Synthesis

Materials and Methods

Reagents and NMR solvents (chloroform-*d*, DMSO-d_6, D_2O (> 99.8 %D)) were purchased from Sigma-Aldrich. ^1H and ^{13}C chemical shifts are reported in ppm after calibration to residual isotopic solvent or tetramethylsilane. Coupling constants (*J*) are reported in hertz (Hz). Thin layer chromatography (TLC) was performed on pre-coated aluminum plates of Silica Gel 60 F254 from Merck. Reactions were monitored by thin layer chromatography with silica gel (visualized by UV-vis lamp, or developed with $KMnO_4$ stain) or directly by UV-vis spectroscopy of diluted reaction aliquots. Column chromatography was carried out using Caledon Silica Gel 60; 50-200 microns 70-300 mesh, or using Sephadex LH-20 with dry bead size of 18-111 µm from GE Health Care. Dialysis was performed with Spectrum Laboratories Float-A-Lyzer G2 500 MWCO.

All the spectroscopy data for structural characterizations were obtained using the research facilities at University of Toronto Scarborough campus (TRACES centre) or at St. George campus (Chemistry department). NMR spectra were recorded on Brucker 500 MHz or Varian Unity 500 MHz spectrometer. Infrared spectra were recorded on a Bruker Alpha FT-IR Spectrometer. High resolution mass spectra were obtained from an ABI/Sciex Qstar mass spectrometer (ESI).

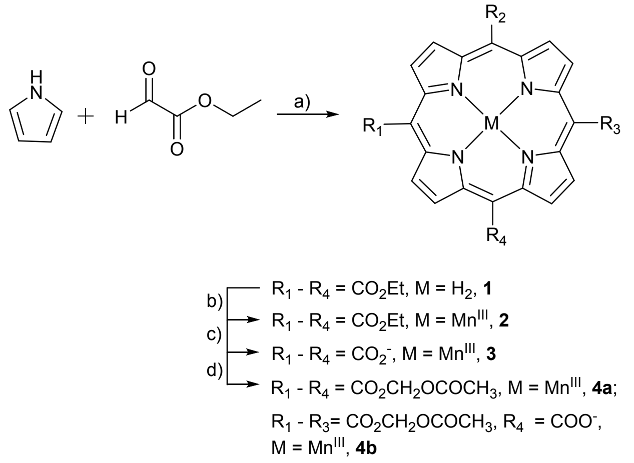

Scheme S1. Synthesis of **4a** and **4b**; reagents and conditions: (a) BF_3OEt_2, DCM, DDQ, 25 °C, 10%; (b) $MnCl_2{\cdot}4H_2O$, DMF, reflux, 85%; (c) NaOH, EtOH/THF, reflux, 85%; (d) DBU, AMBr, DMF, 24 h, 55 °C, 65%.

5,10,15,20-tetrakis(ethoxycarbonyl)porphyrin (1)

The synthesis of **1** was accomplished using a modified literature method.[1] Ethyl glyoxalate (50% in toluene, 1.88 mL, 9.40 mmol) in dichloromethane (DCM) and pyrrole (0.65 mL, 9.40 mmol) were stirred at 25 °C in the dark. After 10 min BF_3•OEt_2 (0.40 mL, 3.10 mmol) was added drop wise. The reaction was stirred at 25 °C for 1.25 h followed by the addition of DDQ (1.60 g, 7.05 mmol). After a stirring period of 2.25 h Et_3N (0.43 mL, 3.06 mmol) was added via syringe and the reaction mixture was concentrated on a rotary evaporator. The crude solution was filtered over Celite using DCM as an elution solvent. The solution was concentrated on a rotary evaporator. Purification by column chromatography (DCM) on silica gel gave 139 mg (10%) of **1** as a black-purple solid. ^1H NMR (500 MHz, $CDCl_3$): δ = 9.52 (8 H, s, por-β), 5.11 (8 H, q, J = 7.2 Hz), 1.81(12 H, t, J = 7.2 Hz), -3.33 (2 H, s, NH); UV-vis (MeOH) λ_{max} = 409 nm.

[5,10,15,20-tetrakis(ethoxycarbonyl)porphyrinato]manganese(III) chloride, (2)

The current step was performed according to the literature method.[1] Compound **1** (18 mg, 29.7 μmol) was dissolved in 2 mL DMF. $MnCl_2$•$4H_2O$ (18 mg, 89.2 μmol) was added and the reaction was refluxed open to air for 5 h followed by 11.5 h at 25 °C. Distillation of DMF resulted in a black-purple solid. Purification by silica gel column chromatography was performed, eluting unreacted **1** with 4% MeOH in DCM and subsequent elution of the product with 7% MeOH in DCM. 17 mg (85%) black-purple solid **2** was isolated. UV-vis (MeOH) λ_{abs} = 328, 366, 387, 413, 456, 552 nm, λ_{max} = 456 nm; IR (neat): v = 2920, 2852 (sp^3 C–H, ethyl alkane), 1715 (sp^2 C=O, carbonyl), 1239, 1195 (C–O single bond, ethyl ester) cm^{-1}; ESI MS found *m/z* = 651.1 ($[M]^+$), calcd for $C_{32}H_{28}MnN_4O_8^+$, *m/z* = 651.1.

[5,10,15,20-tetrakis(carboxy)porphyrinato]manganese(III), (3, MnTCP)

Ethanol (10 mL) and 2 M NaOH (10 mL) were added to a solution of **2**, (14 mg, 21.9 μmol) in THF (6 mL). The reaction was refluxed for 12 h followed by neutralization with 3 M H_2SO_4. Purification by Sephadex LH-20 chromatography with ultrapure water followed by dialysis gave 11.2 mg (85%) of **3** as a dark brown solid. UV-vis (HEPES buffer, IS = 100 mM, pH = 7.0, 25 ^oC) λ_{abs} = 325, 377, 397, 421, 465, 561, 592 nm, λ_{max} = 465 nm; IR (neat): v = 3378 (carboxylic O–H), 1567 (sp^2 C=O, carbonyl), 1383, 1319 (C–O single bond, carboxylic acid) cm^{-1}; ESI MS found *m/z* = 539.0032 ($[M]^+$), calcd for $C_{24}H_{12}MnN_4O_8^+$, *m/z* = 539.0030.

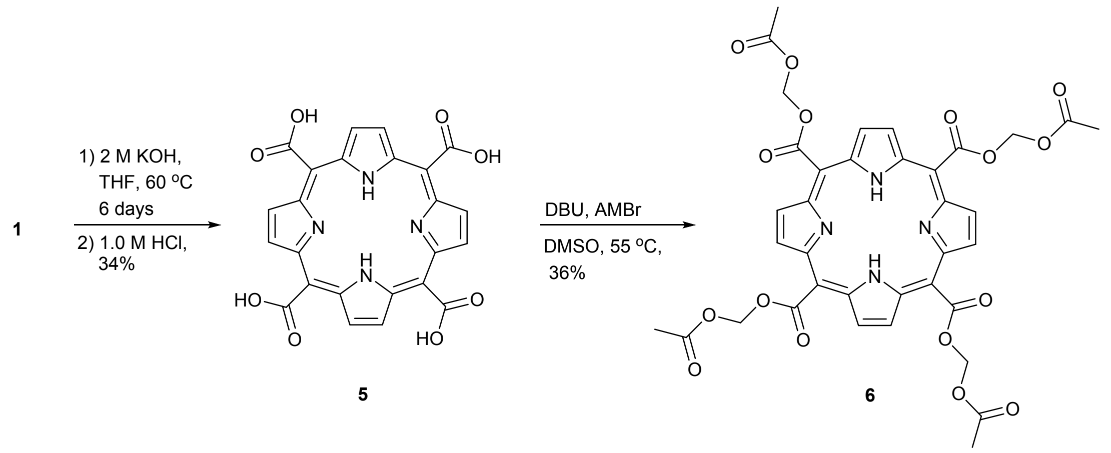

Scheme S2. Synthesis of compound **5** and **6**.

5,10,15,20-tetracarboxyporphyrin, (5, TCP)

To compound **1** (18 mg, 30 µmol) in THF (1.6 mL) was added 2 M KOH (1 mL). The reaction temperature was maintained at 60 °C for 6 days. The aqueous layer was diluted, washed with EtOAc and slowly neutralized with 2 M HCl. The mixture was concentrated in vacuo. Acidification to pH 2.4 with 1 M HCl resulted in precipitation of a dark red solid. Filtration and washing with cold deionized water and cold acetone gave compound **5** as a red solid in 34% yield. UV-vis (HEPES buffer, 25 °C) λ_{max} = 407 nm; IR (neat): ν = 3299 (carboxylic O–H), 1566 (sp^2 C=O, carbonyl), 1350, (C–O single bond, carboxylic acid) cm^{-1}; ^{1}H NMR (500 MHz, DMSO-d_6): δ = 9.68 (8 H, s, por-β), -3.50 (2 H, s, NH); ^{13}C NMR: δ = 177.14 (COO^-), 142.86, 131.02 (β-CH), 119.31 (*meso*-C); ^{13}C DEPT δ = 131.09; ESI MS positive mode found m/z = 487.0 ($[MH]^+$), calcd for $C_{24}H_{15}N_4O_8^+$, m/z = 487.1.

5,10,15,20-tetra(acetoxymethoxycarbonyl)porphyrin, (6, TAMP)

DBU (17 µL, 69 µmol, 4.8 eq.) was added dropwise to a solution of **5** (7 mg, 14.4 µmol) in DMSO (1 mL). After 10 min the first aliquot of acetoxymethyl bromide (5 µL, 34 µmol, 2.4 eq) was added. The reaction was stirred at 25 °C for 2 h then a second aliquot of acetoxymethyl bromide (5 µL, 34 µmol, 2.4 eq) was added. After a total reaction time of 4 h, distillation of DMSO at 70 °C in vacuo

resulted in a crude red oil. The crude material was dissolved in DCM and washed with water, then brine. The organic layer was dried over sodium sulfate, filtered and dried in vacuo. Purification by flash column chromatography (50% EtOAc in hexanes, $R_f = 0.18$) on silica gel gave 3.9 mg (36%) of compound **6** as a red solid. UV-vis (DMSO, 25 °C) λ_{max} = 410 nm, IR (neat): v = 2923, 2852 (sp^3 C–H), 1759, 1713 (sp^2 C=O, carbonyl), 1247, 1180 (C–O single bond, ester) cm^{-1}; ^1H NMR (500 MHz, $CDCl_3$): δ = 9.57 (8 H, s, por-β), 6.64 (8 H, s, $-OCH_2O-$), 2.38 (12 H, s, $-CH_3$), -3.39 (2 H, s, NH); ESI MS positive mode found m/z = 775.2 ([MH^+], calcd for $C_{36}H_{31}N_4O_{16}^+$, m/z = 775.2, Fig. S24-S25).

3. GFAAS

Four standards were prepared at 3 ppb, 9 ppb, 18 ppb and 24 ppb Mn and a linear calibration curve ($r^2 > 0.9995$) was obtained. A sample of MnAMP was prepared at around 12 ppb and the absorbance was measured to determine the accurate Mn content. The purity of the final product was determined to be ≥ 95%. Spectroscopic analyses of the cell digestion samples was determined by the same method.

Temp (°C)	Time (s)	Ramp (°C/s)	Gas Flow (L/min)
100	40.0	10	0.2
1100	20.0	150	0.2
1900	3.0	0	Off
2500	3.0	0	0.2

Table S1. Electrothermal program for GFAAS.

4. LC-MS

The purity of the final product was analyzed and determined to be ≥ 95% pure using analytical HPLC/MS, on an Agilent-Hewlett Packard 1100 series HPLC-MSD system equipped with an Agilent 1100 series diode array UV-vis detector and an Extend C-18 reverse phase column (2.1 mm × 50 mm, 1.8 μm). All compounds were detected at 465 nm and ran through at a flow rate of 0.2 mL/min with MeOH as the organic phase and 10 mM NH_4Ac (pH 6.8) as the aqueous phase. **3** was ran through using 5% MeOH. The HPLC chromatogram of **3** showed a single peak eluted at 0.86 min with 100% purity, corresponding MS found m/z = 539.0 ([M+]). Due to limited solubility MnAMP was dissolved in MeOH and infused into ultrapure water. **4a** was isolated in a trace amount and ran through using gradients in Table S2. The chromatogram confirmed decomposition of **4a** (MS found m/z = 827.0 ([M+]), at 19.1 min), to form the more stable **4b** (MS found m/z = 755.1 ([M+]), at 18.5 min). **4b** was ran through using gradients in table S3, the chromatogram showed a single peak at 17.8 min with 100% purity. The same method was used to monitor the progress of the synthetic step of installing the AM esters on **3**, all intermediates as well as **4a** and **4b** were detected as separated HPLC peaks. The partially hydrolyzed samples from the esterase reaction were also ran through using the same gradients, and the results are summarized in Table S4. All intermediates were separated from **4b**. The ratio of **4a**/**4b** (referred to collectively as MnAMP) used for all enzyme and cell experiments was determined using the same gradients in Table S3 and the chromatogram showed two peaks in a 53:47 ratio, **4a** at 20.4 min and **4b** at 19.3 min.

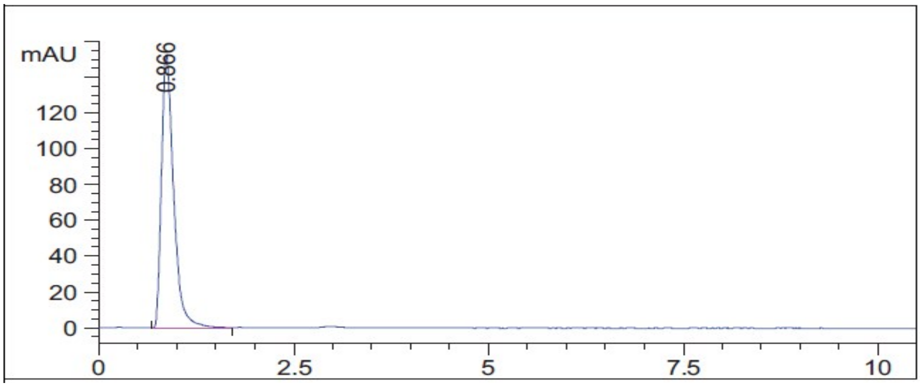

Figure S1. HPLC chromatogram of **3**, elution occurred at 0.9 min with 100% purity.

Time (min)	0	3	20
MeOH:H_2O	5:95	5:95	100:0

Table S2. Solvent gradients used for separation of **4a** and **4b**.

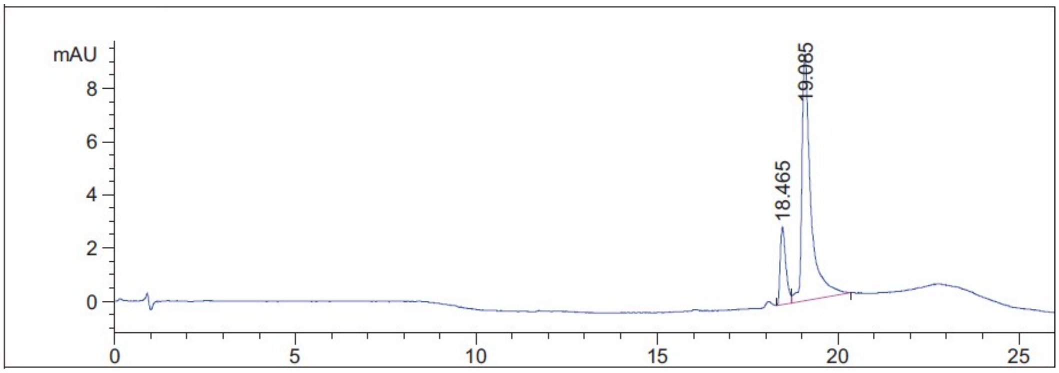

Figure S2. HPLC chromatogram of 4a isolated by preparative TLC. Elution occurred at 19.1 min **4a** (83%) and 18.5 min **4b** (17%).

Time (min)	0	3	12	14	20
MeOH:H_2O	5:95	5:95	70:30	70:30	100:0

Table S3. HPLC-MS solvent gradients.

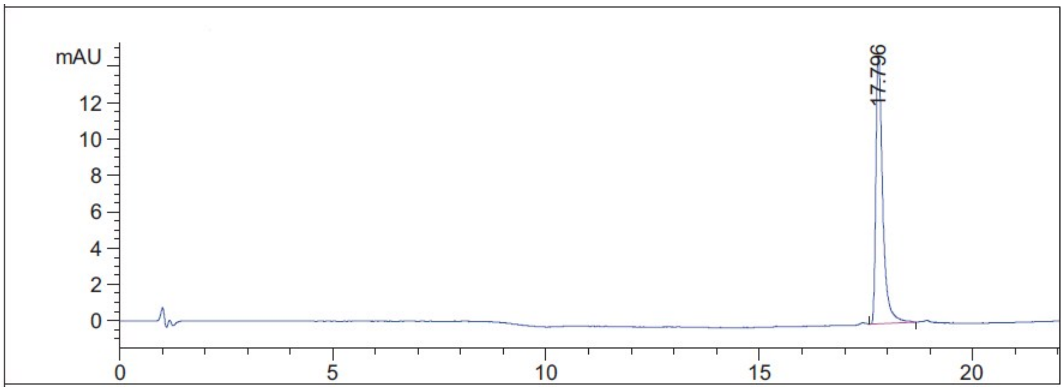

Figure S3. HPLC chromatogram of **4b**. Elution occurred at 17.8 min with 100% purity.

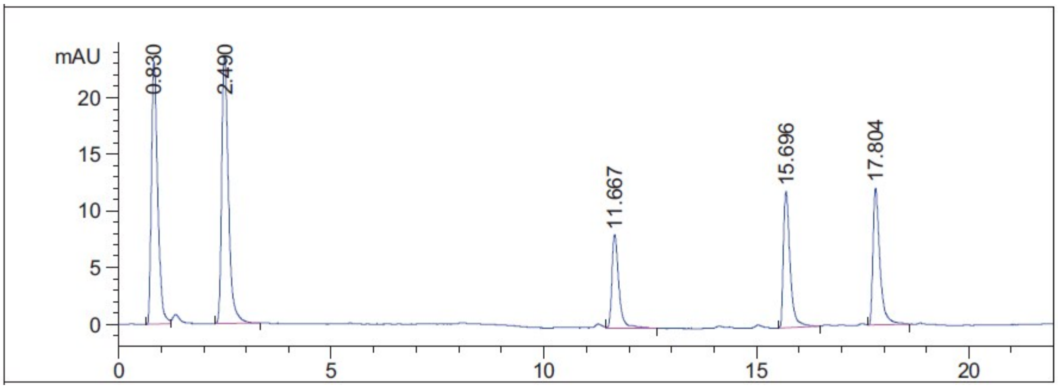

Figure S4. HPLC chromatogram of 4b and the hydrolysis products MnBiAMP + regioisomer, MnMAMP, **3**.

Compound	Formula	Calculated (*m/z*)	Retention Time (min)	MSD (*m/z*)
MnTriAMP (**4b**)	$C_{33}H_{24}MnN_4O_{14}^+$	755.07	17.804	755.0 $[M]^+$ $(C_{33}H_{24}MnN_4O_{14})^+$
MnBiAMP + regioisomer	$C_{30}H_{20}MnN_4O_{12}^+$	683.05	15.696<br>11.667	683.0 $[M]^+$ $(C_{30}H_{20}MnN_4O_{12})^+$
MnMAMP	$C_{27}H_{16}MnN_4O_{10}^+$	611.02	2.490	611.0 $[M]^+$ $(C_{27}H_{16}MnN_4O_{10})^+$
MnTCP (**3**)	$C_{24}H_{12}MnN_4O_8^+$	539.00	0.830	539.0 $[M]^+$ $(C_{24}H_{12}MnN_4O_8)^+$

Table S4. HPLC-MS separation of intermediates.

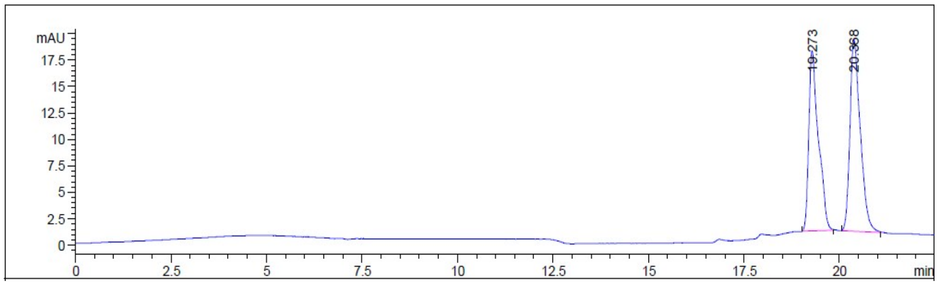

Figure S5. HPLC chromatogram to determine product ratio for **4a**:**4b** detected at 465 nm. Elution of **4a** occurred at 20.4 min (53%) and elution of **4b** at 19.3 min (47%).

5. Aggregation of MnAMP

Dynamic light scattering experiments were performed on a Malvern Zetasizer Nano ZS instrument at a backscattering angle of 173°. MnAMP (final concentration: 60 µM) in 10 mM HEPES buffer, (IS = 0.1 M, pH = 7.4) was left to aggregate at 25 °C. The absorbance was measured over 8 h. The aggregation was also monitored over 2 h by dynamic light scattering (DLS) to determine the size of the aggregates. The aggregation can be reversed by the addition of 20 µL acetone seen as an increase in absorbance.

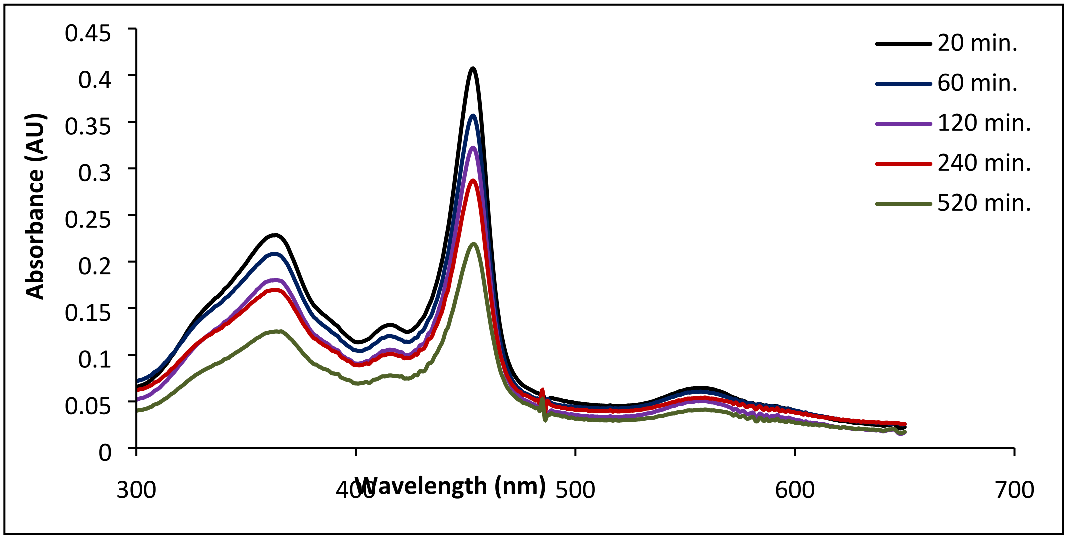

Figure S6. Real time UV-vis absorbance change of MnAMP due to aggregation in HEPES buffer at pH 7.4, 25 °C.

(a)
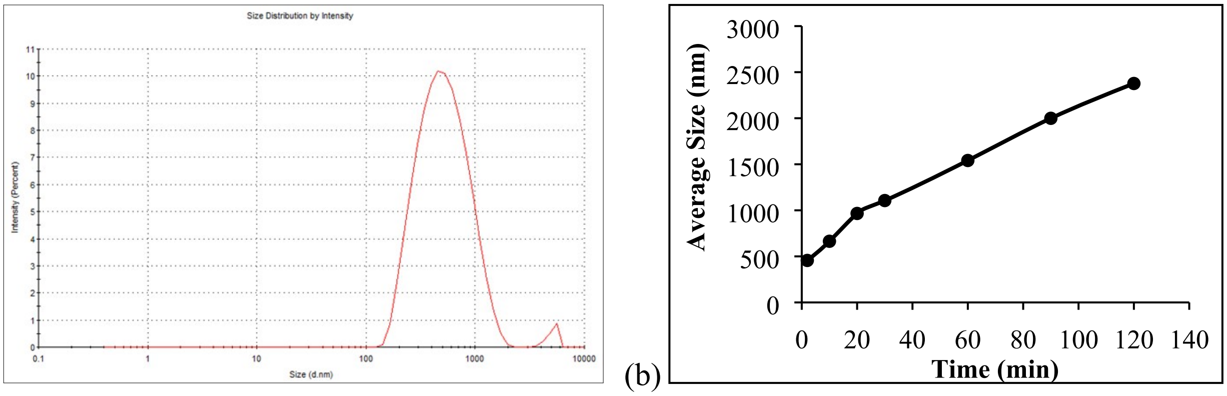

Figure S7. (a) Size distribution of aggregates after 2 min in HEPES buffer measured by DLS. (b) Change in average size of aggregates over time as measured by DLS, in HEPES buffer pH 7.4, 25 °C.

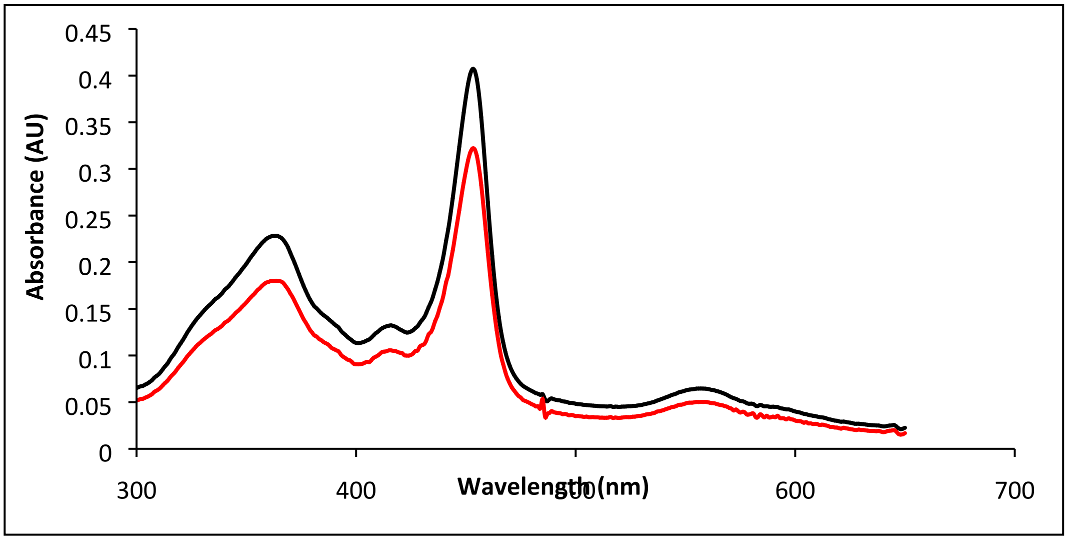

Figure S8. UV-vis absorbance of MnAMP in HEPES buffer pH 7.4, after 2 h in the dark (red) and after addition of 20 μL acetone (black) at 25 °C.

6. Esterase Hydrolysis

Esterase Hydrolysis Monitored by UV-visible Spectroscopy

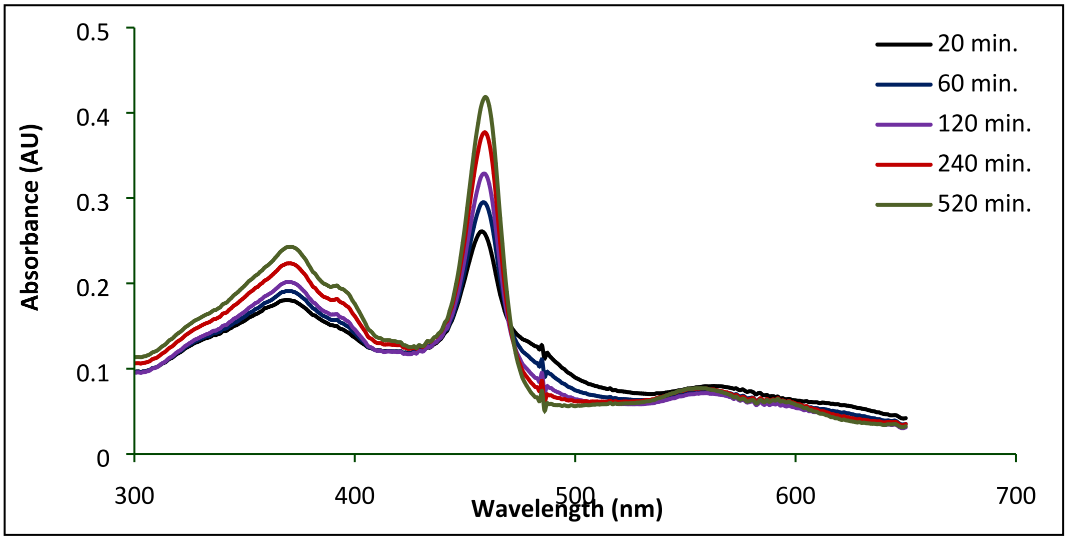

Figure S9. Real time UV-vis absorbance change of MnAMP via esterase catalyzed hydrolysis in HEPES buffer at pH 7.4, 25 °C.

Esterase Hydrolysis Monitored by HPLC

The esterase hydrolysis study was monitored using analytical HPLC, on an Agilent 1100 system equipped with an Agilent 1100 series diode array UV/vis detector and an Eclipse C-18 reverse phase column (4.6 mm × 150 mm, 5 μm). All compounds were detected at 465 nm and ran through at a flow rate of 0.8 mL/min with MeOH as the organic phase and 10 mM NH_4Ac (pH 6.8) as the aqueous phase. In order to monitor the esterase reaction a method was developed to separate **4a** (MnTAMP) and **4b** (MnTriAMP) from the intermediates and the fully hydrolyzed product, **3** (MnTCP). Gradient sequence: 0-3 min constant at 5% MeOH; 3–10 min linear gradient from 5% to 80% MeOH; 10–16 min linear gradient from 80% to 90% MeOH. With this method the HPLC chromatogram of **3** showed a single peak eluted at 1.6 min with 100% purity. HPLC chromatogram of **4b** showed a single peak at 12.3 min at 100%. MnAMP was run with the same method and the chromatogram showed two peaks, **4a** at 12.6 min and **4b** at 12.3 min. The same method was used

to monitor the progress of the esterase reaction and the chromatogram showed all intermediates were detected as separated peaks.

Esterase hydrolysis was carried out with MnAMP (final concentration: 940 μM) in 50 mM HEPES buffer, (IS = 0.5 M, pH = 7.4) at 25 °C, with porcine liver esterase (3.1.1.1) at 20 Units/mL activity. Immediately the substrate began to aggregate. Upon addition of esterase the reaction and control were placed on a mechanical shaker (400 rpm). HPLC samples were worked up by removing 50 μL aliquots, adding 50 μL cold CH_3CN, centrifuging at 14,000 rpm and diluting 50 μL of the supernatant into 1150 μL water and passing the solution through 0.45 μm nylon filters. The control and esterase reaction were monitored for 24 h. The results are summarized in Table S5.

Compound	MnTAMP	MnTriAMP	MnBiAMP	MnBiAMP	MnMAMP	MnTCP
Retention time (min)	12.58	12.30	10.99	9.53	7.20	1.58
Product ratio before enzyme addition	53	47	0	0	0	0
Product ratio at 15 min	0	69.4	0	30.6	0	0
Product ratio at 2 h	0	52.4	0	47.6	0	0
Product ratio at 4 h	0	51.2	0	48.8	0	0
Product ratio at 24 h	0	7.7	4.4	37.4	32.3	18.2

Table S5. Esterase hydrolysis monitored by HPLC; % area determined for MnTAMP, MnTriAMP, hydrolysis intermediates and MnTCP at 15 min, 2, 4 and 24 h after addition of esterase.

Esterase Hydrolysis Monitored by Relaxivity

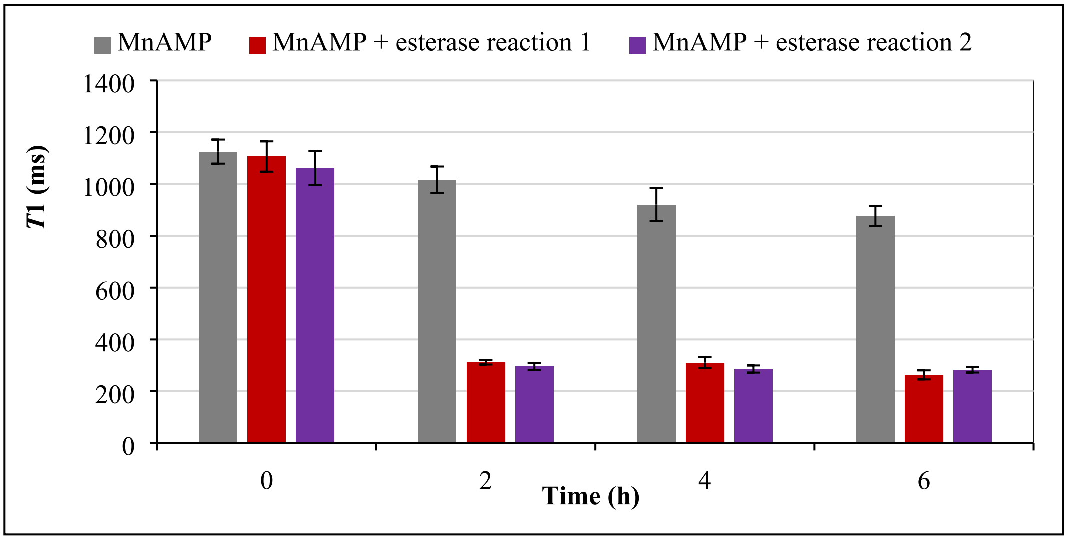

Figure S10. Relaxation times, $T_{1,\ obs}$ (ms) of MnAMP solutions in the presence and absence of porcine liver esterase at 1.5 T, 25 °C, $T_{1,\ d}$ = 2568 ± 17 s, (PBS), data was acquired by relaxometry by means of standard inversion recovery techniques, fitted by non-linear regression with SD from the fitting.

7. Cell Labeling, Viability and MRI

Human Glioma U373 Cell Line

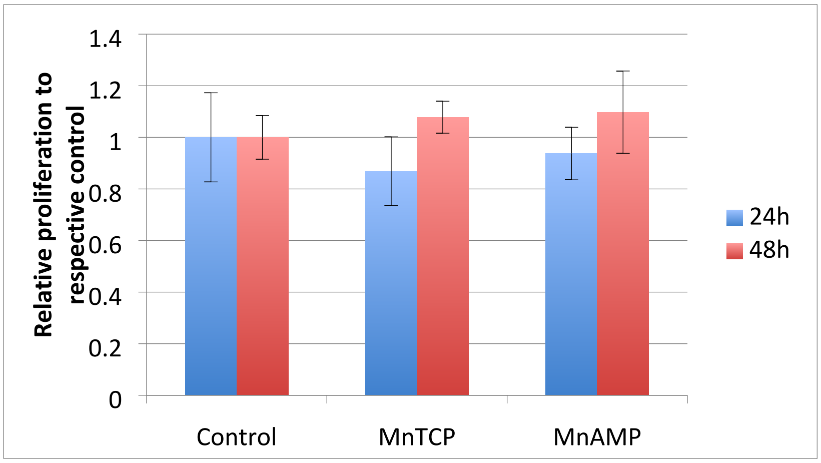

Figure S11. Results from MTT cell proliferation assay. The error bars represent the SD among triplicate measurements. Results were statistically analyzed with a 1-way ANOVA and Tukey post hoc test. No statistical differences were found between the groups.

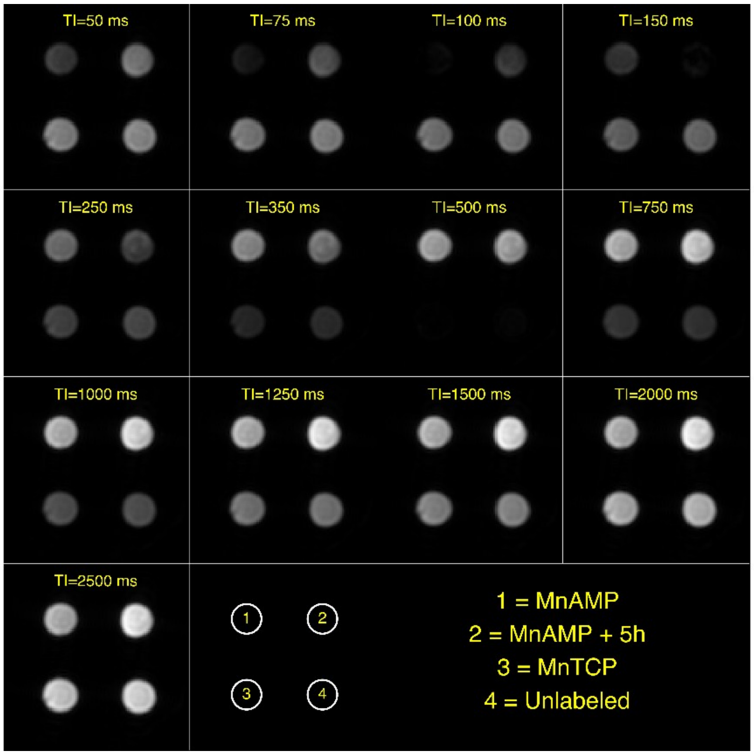

Figure S12. Inversion recovery fast spin echo images of MnAMP labeled cells, MnAMP labeled cells with 5 h in fresh media, MnTCP labeled cells and unlabeled control cells at 3 T, room temperature, acquired at different inversion times.

Sample	Experiment 1	Experiment 2	Experiment 1	Experiment 2
	T_1 at 1 T^a (ms)	T_1 at 1 T^a (ms)	T_1 at 3 T^b (ms)	T_1 at 3 T^b (ms)

Control	969 ± 69	889 ± 56	1134 ± 18	1096 ± 19
MnAMP	95 ± 11	85 ± 7	161 ± 4	171 ± 5
MnAMP 5 h[c]	147 ± 17	163 ± 11	272 ± 12	209 ± 13
MnTCP	879 ± 10	878 ± 12	1048 ± 15	1025 ± 17

Table S6. T_1 of U373 cells labeled with MnAMP, MnAMP after 5 h in medium and MnTCP, the experiment was duplicated. [a]Data at 1 T was acquired by relaxometry by means of standard inversion recovery techniques, fitted by non-linear regression with SD from the fitting; [b]Data at 3 T was quantified from the MR images based on a pixel-by-pixel relaxation time analysis, the SD represents the variation among the pixels; [c]Cells labeled with MnAMP were grown in MnP-free medium for extra 5 h.

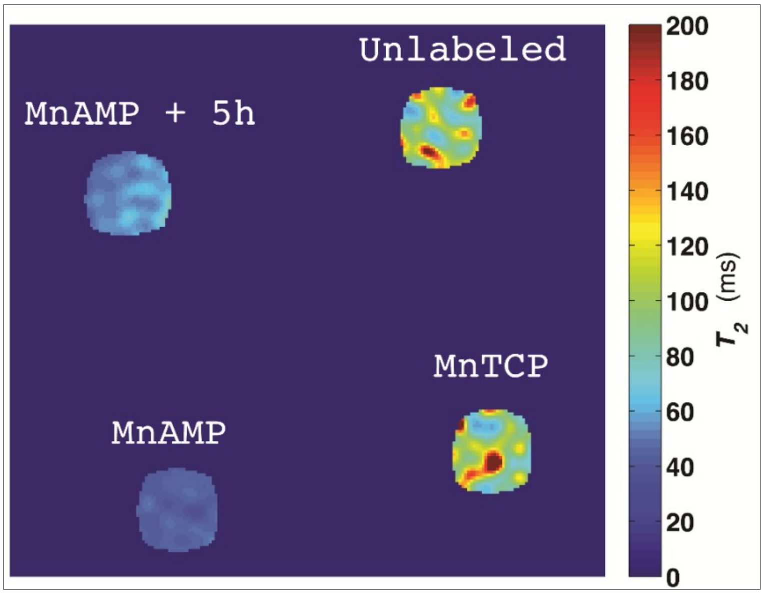

Figure S13. T_2 maps of MnAMP labeled cells, MnAMP labeled cells with 5 h in fresh media, MnTCP labeled cells and unlabeled control cells at 3 T, room temperature, obtained from Spin-Echo MR Images.

Cell Lysis, HPLC and UV-Visible Spectroscopy

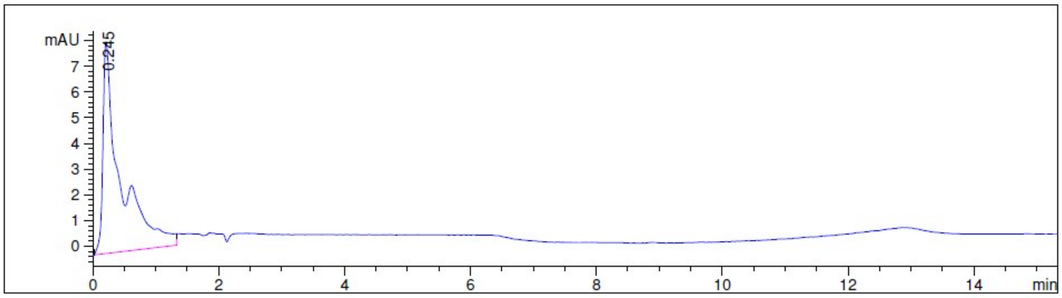

Figure S14. HPLC chromatogram of the cytosolic fraction of the unlabeled cells after cell lysis.

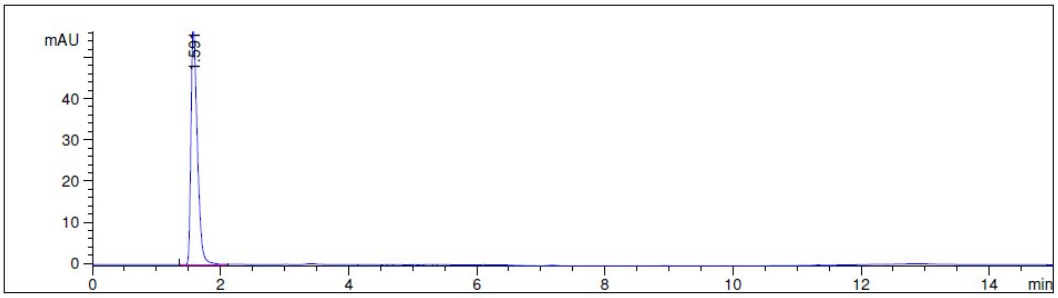

Figure S15. HPLC chromatogram of cytosolic fraction after MnAMP labeling and cell lysis confirms complete intracellular conversion of MnTAMP, MnTriAMP, MnBiAMP and MnMAMP to MnTCP present at 1.59 min.

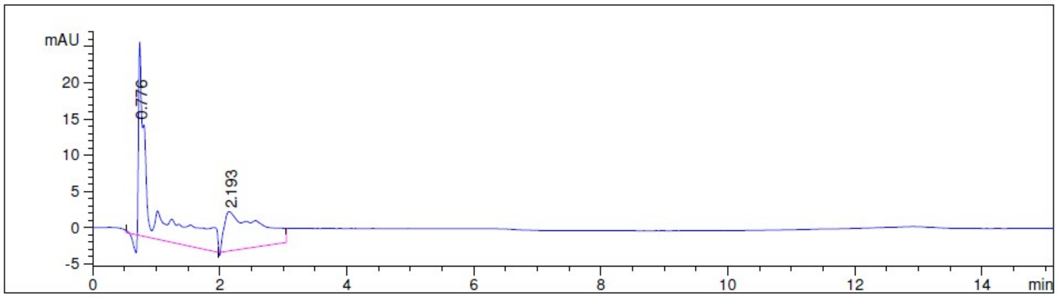

Figure S16. HPLC chromatogram of nuclear fraction of the unlabeled cells after cell lysis.

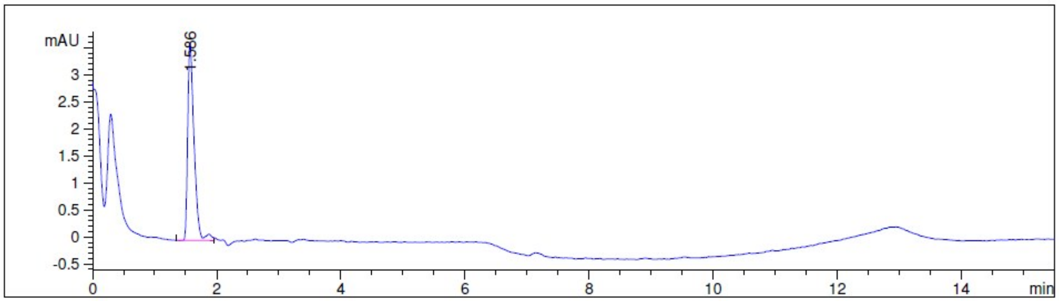

Figure S17. HPLC chromatogram of nuclear fraction after MnAMP labeling and cell lysis. MnTCP present at 1.59 min. (6% MnTCP relative to concentration in cytosolic fraction).

(a) 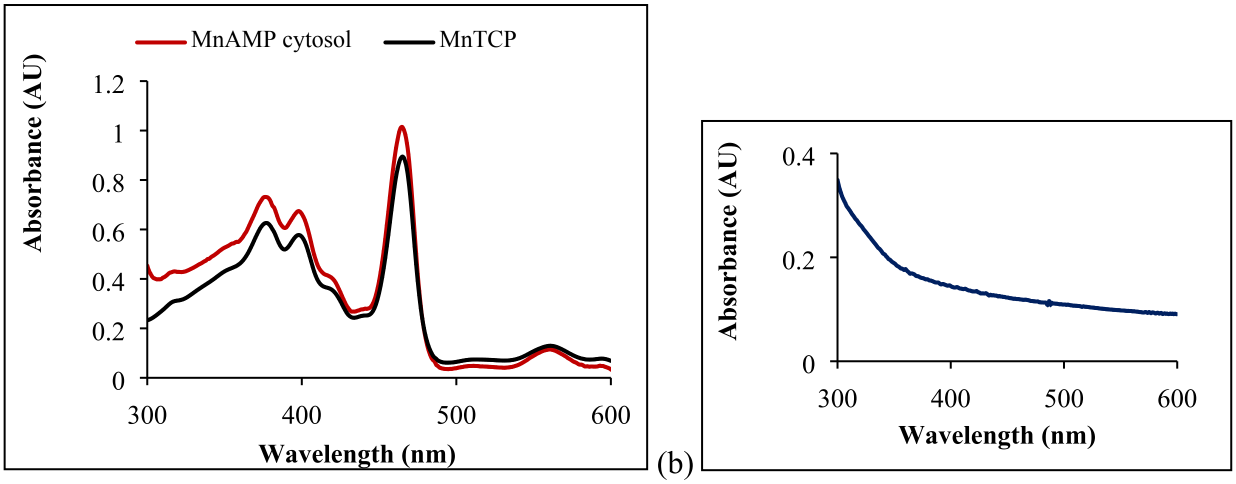

(b)

Figure S18. (a) UV-vis absorbance of cytosolic fraction after MnAMP labeling (red) and absorbance of MnTCP (black) for comparison. (b) UV-vis absorbance of cytosolic fraction from the unlabeled control cells. All spectra recorded at 25 °C, the cytosol of the unlabeled control cells was used as a blank for the measurement of MnAMP cytosol. PBS was used as a blank for the unlabeled control cytosol.

MDA-MB-231 Cell Line

Human breast cancer cells MDA-MB-231 were grown at 37 °C with 5% CO_2 in 1640-RPMI medium supplemented with 10% fetal bovine serum and 0.5% penicillin streptomycin. Contrast agents were quantified for manganese content by AAS prior to cell labeling. MnTCP was dissolved in ultrapure water and MnAMP was dissolved in DMSO to give 17 mM stock solutions. The stock solutions were added to the medium with the cells resulting in 80 μM incubation of MnAMP or MnTCP for 2 h. The medium was removed and the cells were washed with fresh medium 3 times and then were harvested by addition of 0.05% trypsin EDTA followed by washing with PBS to detach the cells. Viability was assessed directly after labeling with trypan blue exclusion test and observation of cell morphology by phase microscopy following the previous procedure.[2] The results are listed in Table S7. For MRI, the cell pellets were formed by centrifugation at 440 g for 10 min. Subsequent MR was done with a T_1-weighted 2D spin-echo image: TR = 100 ms, TE =

14.163 ms, 3 mm slice thickness, 0.5 × 0.5 mm in-plane resolution. The signal intensity was analyzed to give the relaxation times T_1 and T_2 of the cell pellets summarized in Table S8.

Treatment	Viability (%) (± SEM)
Control	98 ± 2
MnTCP	98 ± 1
MnAMP	97 ± 2

Table S7. Viability measurements for MDA-MB-231 breast cancer cell pellets: directly after incubation.

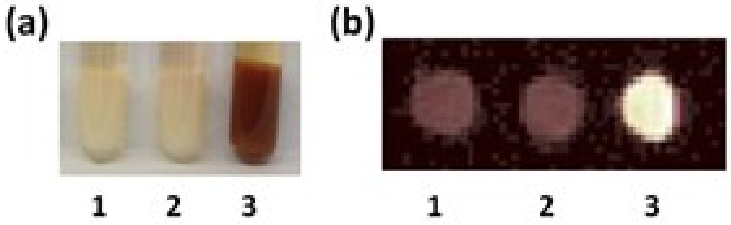

Figure S19. (a) Photograph of cell pellets prior to MRI: from left untreated cells (1), cells treated with MnTCP (2) and MnAMP (3); (b) the corresponding MR image of same cells at 3 T and 25 °C. TR = 100 ms, TE = 14.163 ms, 3 mm slice thickness, 0.5 × 0.5 mm in-plane resolution.

	T_1 (ms)	T_2 (ms)
Untreated cells	1107.3 ± 26.6	108.6 ± 9.5
Cells treated with MnTCP	1072.1 ± 28.0	108.1 ± 9.3
Cells treated with MnAMP	215.4 ± 12.1	66.7 ± 4.4

Table S8. T_1 and T_2 determined from MDA-MB-231 breast cancer cell pellets, untreated or treated with MnTCP or MnAMP.

Cell Digestion

To the cell pellet was added 1.0 ml 70% HNO_3. The mixture was sonicated at 40 °C for 7 h. Following dissolution of the cells, the samples were diluted with water and Mn content was determined by GFAAS. To the cell membrane fraction was added 300 µL 70% HNO_3. The mixture

was sonicated at 40 °C for 7 h. Following dissolution of the cells, the samples were diluted with water and Mn content was determined by GFAAS.

8. Structural Characterization

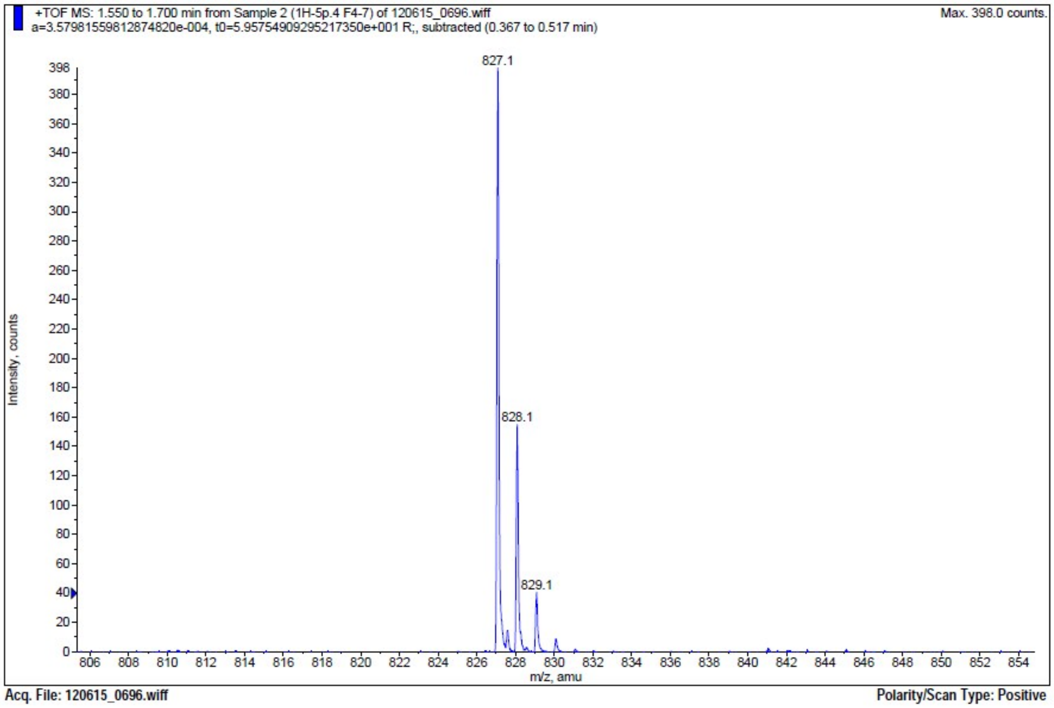

Figure S20. HR ESI-MS positive mode *m/z* = 827.0875, [M^+] for **4a**.

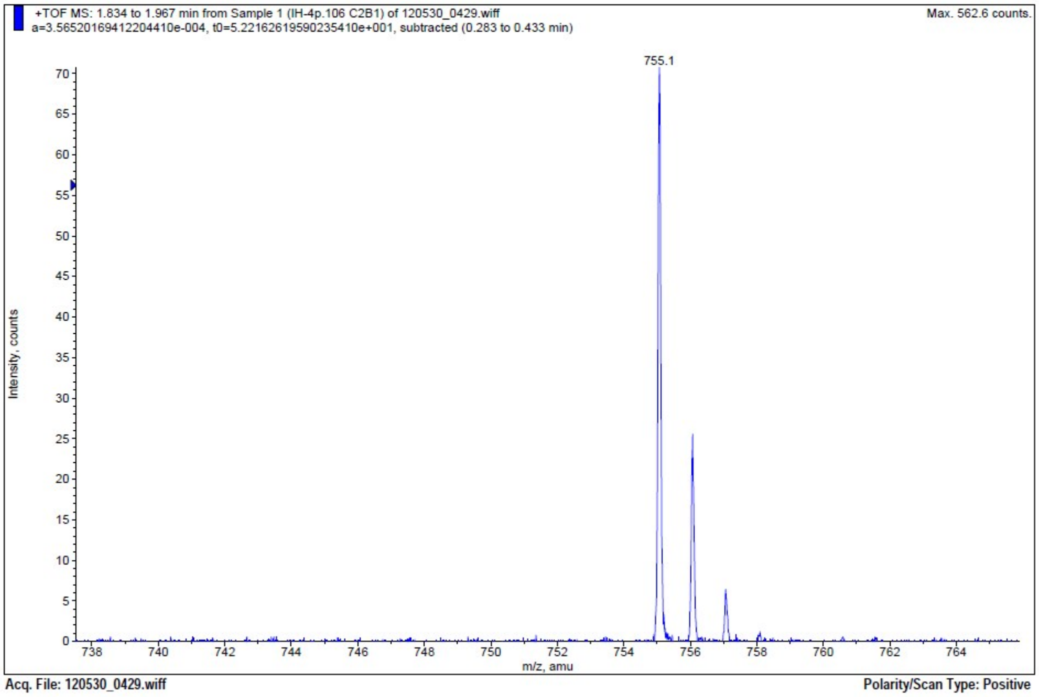

Figure S21. High resolution ESI-MS positive mode *m/z* = 755.0678, [M^+] for **4b**.

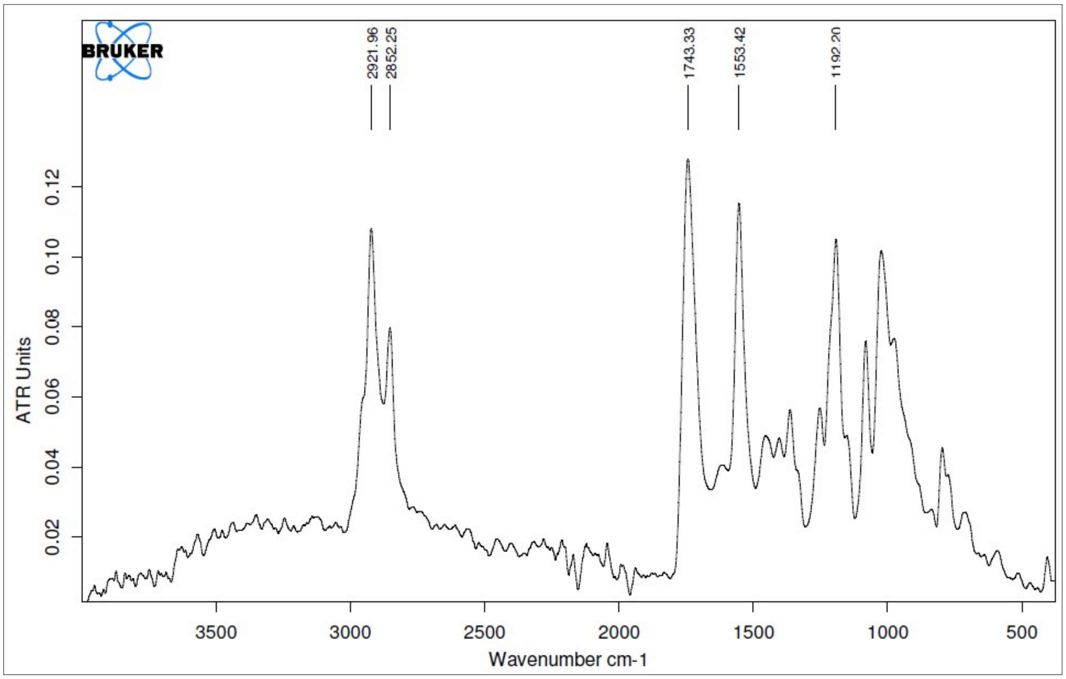

Figure S22. FTIR spectrum of **4b** (neat). The relevant absorptions are: v = 2921, 2852 (sp^3 C-H, methyl alkane), 1743 (sp^2 C=O, carbonyl), 1553 (sp^2 COO$^-$), 1192 (C–O single bond, ester).

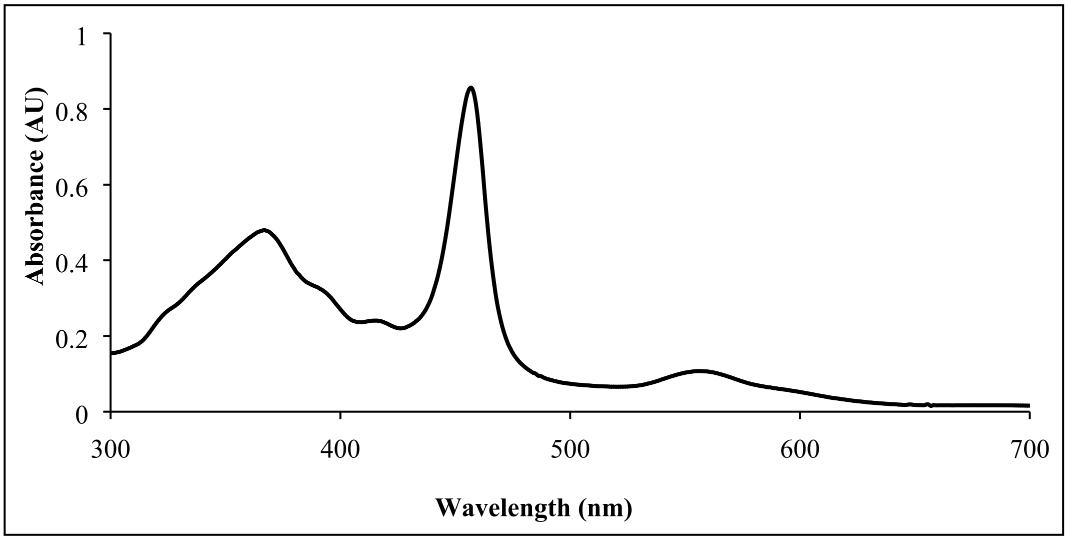

Figure S23. UV-vis absorbance of **4b**, in HEPES buffer, pH 7.4, 25 °C.

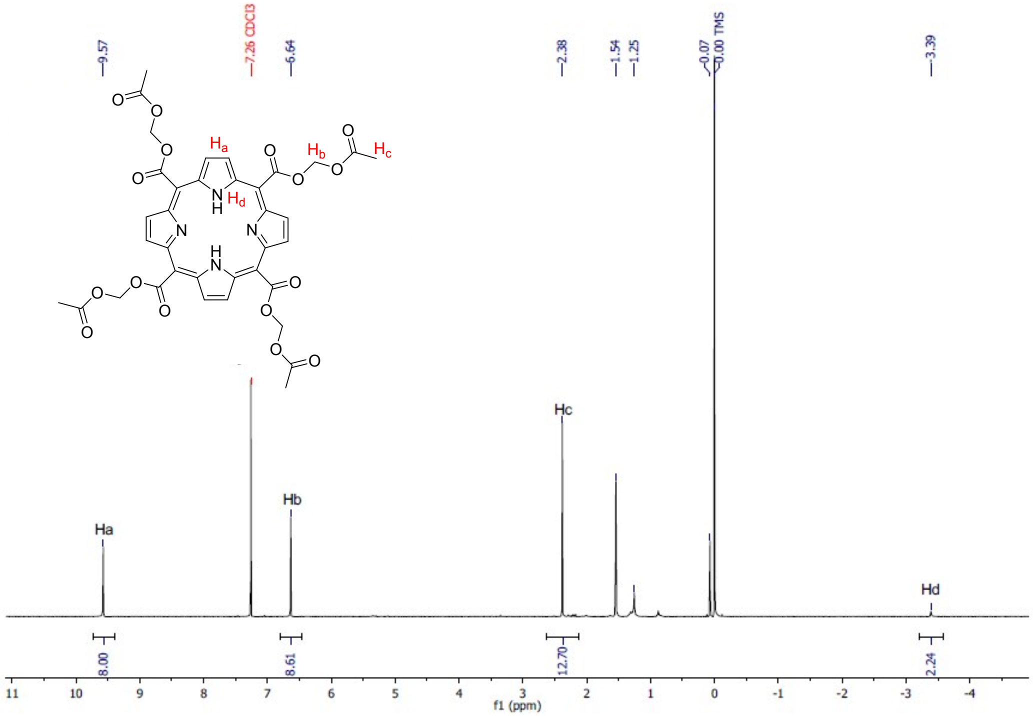

Figure S24. ^{1}H NMR spectrum of **6** in $CDCl_3$. The residual solvent peaks are chloroform (7.26 ppm), water (1.54 ppm), hydrogen grease (1.25 ppm) and silicone grease (0.07 ppm).

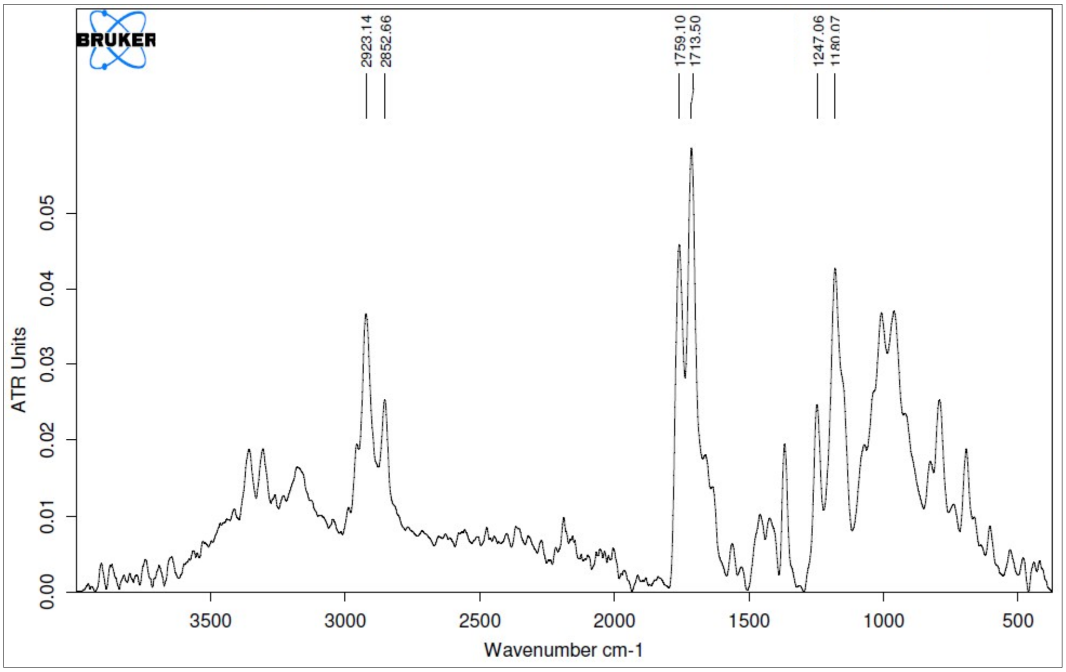

Figure S25. IR spectra of **6** (neat). The relevant absorptions are: ν = 2923, 2852 (sp^3 C–H, methyl alkane), 1759, 1713 (sp^2 C=O, carbonyl), 1247, 1180 (C–O single bond, ester) cm^{-1}.

9. References

(1) Trova, M. P.; Gauuan, P. J. F.; Pechulis, A. D.; Bubb, S. M.; Bocckino, S. B.; Crapo, J. D.; Day, B. J. *Bioorg. Med. Chem.* 2003, **11**, 2695.

(2) Nofiele, J. T.; Cheng, H. L. M. *PLoS One* 2013, **8**, e58617.
